# Supplementary material for: Sociodemographic and mental health predictors of mental health service use across provider types
Source: PLoS One. 2026 May 18;21(5):e0326556. doi: 10.1371/journal.pone.0326556 (PMC13183218; doi:10.1371/journal.pone.0326556)
Supplement: S1 File — (PDF) [file pone.0326556.s001.pdf]

Appendix 1: Variable Definitions and Availability Across CCHS Cycles (2007–2020)

**Construct**	**Cycles Available**	**Cross Cycle Differences**	**Reason for 2019-2020 Restriction**
Sex	2007-2008; 2009-2010; 2011-2012; 2013-2014; 2015-2016; 2017-2018; 2019-2020		
Age	2007-2008; 2009-2010; 2011-2012; 2013-2014; 2015-2016;2017-2018; 2019-2020	Age groups different (2007-2008, 2009-2010, 2011-2012, 2013-2014, 2015-2016, 2017-2018)	Age grouped differently in all other cycles
Household Income	2013-2014; 2015-2016; 2017-2018; 2019-2020	Measured in distribution in 2007-2008, 2009-2010; 2011-2012	Income measured differently between 2007-2012
Education	2013-2014; 2015-2016; 2017-2018; 2019-2020	Missing variable in 2007-2008, 2009-2010, 2011-2012	Education not measured until 2013-2014 cycle
Visible Minority	2019-2020	Missing variable in 2007-2008, 2009-2010, 2011-2012, 2013-2014, 2015-2016, 2018-2019	Visible minority not included until 2019-2020 cycle
Aboriginal Identity	2015-2016; 2017-2018; 2019-2020	Missing variable in 2007-2008, 2009-2010, 2011-2012, 2013-2014	Aboriginal identity not included until 2015-2016 cycle
Immigrant	2007-2008; 2009-2010; 2011-2012; 2013-2014; 2015-2016; 2017-2018; 2019-2020		
Perceived Health	2007-2008; 2009-2010; 2011-2012; 2013-2014; 2015-2016; 2017-2018; 2019-2020		
Perceived Mental Health	2007-2008; 2009-2010; 2011-2012; 2013-2014; 2015-2016; 2017-2018; 2019-2020		

Past 12 Month Contact for Mental Health: Social Worker	2007-2008; 2009-2010; 2011-2012; 2013-2014; 2015-2016; 2017-2018; 2019-2020		
Past 12 Month Contact for Mental Health: Family Doctor	2007-2008; 2009-2010; 2011-2012; 2013-2014; 2015-2016; 2017-2018; 2019-2020		
Past 12 Month Contact for Mental Health: Psychiatrist	2007-2008; 2009-2010; 2011-2012; 2013-2014; 2015-2016; 2017-2018; 2019-2020		
Past 12 Month Contact for Mental Health: Psychologist	2007-2008; 2009-2010; 2011-2012; 2013-2014; 2015-2016; 2017-2018; 2019-2020		
