# Supplementary material for: Sociodemographic and mental health predictors of mental health service use across provider types
Source: PLoS One. 2026 May 18;21(5):e0326556. doi: 10.1371/journal.pone.0326556 (PMC13183218; doi:10.1371/journal.pone.0326556)
Supplement: Attachment — Submitted filename: PONE-D-25-27752_R1 16 March.pdf [file pone.0326556.s003.pdf]

PLOS ONE

# Sociodemographic and Mental Health Predictors of Mental Health Service Use Across Provider Types

--Manuscript Draft--

| | |
|---|---|
| **Manuscript Number:** | PONE-D-25-27752R1 |
| **Article Type:** | Research Article |
| **Full Title:** | Sociodemographic and Mental Health Predictors of Mental Health Service Use Across Provider Types |
| **Short Title:** | Sociodemographic and Mental Health Predictors of Mental Health Service Use Across Provider Types |
| **Corresponding Author:** | Nelson Pang<br>University of Toronto Factor-Inwentash Faculty of Social Work<br>Toronto, Ontario CANADA |
| **Keywords:** | Mental Health Care Access; sociodemographic disparities; Health Care Access: Psychiatric Care; Health Service Trends |
| **Abstract:** | Objective: To examine trends in mental health service use across four provider types (family doctors, psychiatrists, psychologists, and social workers) and identify sociodemographic predictors of provider-specific access in Canada.<br>Methods: This study analyzes seven cycles (2007–2020) of the Canadian Community Health Survey, a nationally representative cross-sectional survey. Trends over time were examined using weighted proportions and counts of service users. Weighted multivariable logistic regression models were applied to the 2019–2020 cycle to assess associations between sociodemographic factors and provider-specific service use.<br>Results: Family doctors were consistently the most accessed providers for mental health concerns, followed by psychologists and social workers, with psychiatrists being least accessed. Psychologist and social worker use increased between 2017 and 2019. In the adjusted regression models (2019–2020), women had higher odds of using family doctors (AOR = 1.21, 95% CI: 1.05–1.39) and social workers (1.19, 1.02–1.40) and lower odds of psychiatrists (AOR = 0.66, 95% CI:0.55–0.79) than men. Adults 65+ had greater odds of family-doctor use (AOR = 4.82, 95% CI: 3.59–6.47) and lower odds of psychologist (AOR = 0.33, 95% CI: 0.24–0.45) and social-worker use (AOR = 0.21, 95% CI:0.16–0.29) than ages 12–17. Post-secondary education (vs less than secondary school) was associated with higher psychologist use (AOR = 1.83, 95% CI 1.12–2.98). Higher income (≥$80,000 vs <$20,000) was associated with lower psychiatrist use (AOR = 0.66, 95% CI 0.50–0.86). Non-Indigenous respondents more often used psychologists (AOR = 1.58, 95% CI: 1.13–2.23), and respondents who are not a visible minority more often used family doctors (AOR = 1.37, 95% CI:1.06–1.77).<br>Conclusion: This study reveals a stratified mental health care system in Canada, where sociodemographic factors shape who accesses which providers. While primary care dominates, growth in psychologist and social worker use suggests shifting patterns of engagement. Findings underscore the need for policies that address financial and structural barriers, promote equitable access, and expand coverage for community-based mental health providers. |
| **Order of Authors:** | Nelson Pang |
| | Jessie Yeung |
| **Response to Reviewers:** | Response to Reviewer for Manuscript: Sociodemographic and Mental Health Predictors of Mental Health Service Use Across Provider Types<br><br>Reviewer #1<br>Reviewer Comment: Title: I suggest the title to be revised to “Mental Health Predictors of Mental Health Service Use Across Provider Types”.<br>Author Response: Thank you for the suggestion. We have opted to use the original title, “Sociodemographic and Mental Health Predictors of Mental Health Service Use Across Provider Types” as it more accurately reflects the scope of the study. In addition to mental health characteristics, our analysis includes a range of sociodemographic variables (e.g., age, gender, income, education, Indigenous identity, |

Powered by Editorial Manager® and ProduXion Manager® from Aries Systems Corporation

and immigration status) as key predictors.

Reviewer Comment: Abstract: Remove the last part of methodology.
Author Response: Thank you for the suggestion. We have removed the last part of methodology from the abstract.

Reviewer Comment: Abstract: Revise the results section in abstract for presenting the regression analysis.

Author Response: The results in the abstract have been edited when presenting the regression analysis and now reads: In the adjusted regression models (2019–2020), women had higher odds of using family doctors (AOR = 1.21, 95% CI: 1.05–1.39) and social workers (1.19, 1.02–1.40) and lower odds of psychiatrists (AOR = 0.66, 95% CI:0.55–0.79) than men. Adults 65+ had greater odds of family-doctor use (AOR = 4.82, 95% CI: 3.59–6.47) and lower odds of psychologist (AOR = 0.33, 95% CI: 0.24–0.45) and social-worker use (AOR = 0.21, 95% CI:0.16–0.29) than ages 12–17. Post-secondary education (vs less than secondary school) was associated with higher psychologist use (AOR = 1.83, 95% CI 1.12–2.98). Higher income (≥$80,000 vs <$20,000) was associated with lower psychiatrist use (AOR = 0.66, 95% CI 0.50–0.86). Non-Indigenous respondents more often used psychologists (AOR = 1.58, 95% CI: 1.13–2.23), and respondents who are not a visible minority more often used family doctors (AOR = 1.37, 95% CI:1.06–1.77).

Reviewer Comment: Introduction: Adding reference for middle of the second paragraph.
Author Response: Thank you for the suggestion. We have added a reference for the middle of the second paragraph.

Reviewer Comment: Provide a research gap and problem statement.
Author Response: We have revised this section to provide a cleaer research gap and problem statement. This section now reads as: A limitation in this research however is that most studies aggregate all types of mental health providers into one variable limiting the examination of differences in who accesses which services and under which circumstances. As a result, we lack nuanced understanding of how sociodemographic factors shape engagement with specific provider types, such as psychologists or social workers, whose roles and funding structures differ significantly from psychiatrists and family doctors. Furthermore, few studies have examined national level trends over time in provider specific mental health service use limiting our ability to assess how patterns have shifted in response to evolving service landscapes, policy changes, or public attitudes towards mental health. To address these gaps, this study focuses on examining how sociodemographic and mental health characteristics are associated with mental health service use across four provider types (family doctors, psychiatrists, psychologists, and social workers) and trends in their use over time in Canada. By disaggregating service use by provider type, this research aims to uncover distinct access patterns across sociodemographic groups and inform efforts to promote equity in mental health care.

Reviewer Comment: Design of the study need to be mentioned the first.
Author Response: We now open the Methods with a clear statement of design and data source and now reads: We conducted a ~~cross-sectional,~~ population-based analysis using seven cycles (2007-2020) of the Canadian Community Health Survey (CCHS) public-use microdata. The CCHS is a nationally representative cross-sectional survey administered by Statistics Canada. The CCHS is a self-reported survey on health, healthcare utilization, and health determinants of the Canadian population aged 12 and above. The CCHS covers the population 12 years of age and over who live in Canada. Excluded from the CCHS are institutionalized residents, full-time members of the Canadian Armed Forces, residents of certain remote regions, or people living on reserves/other Indigenous settlements in the provinces. Altogether, these exclusions represent less than 3% of the target population. Sample sizes varied across cycles, and all analyses applied survey weights provided by Statistics Canada to ensure representativeness of the Canadian population. This study summarized trends of provider specific mental health access across 2007–2020 and provider specific logistic

Powered by Editorial Manager® and ProduXion Manager® from Aries Systems Corporation

regressions were restricted to 2019–2020 because it was the most recent cycle and contained key predictors specified a priori for multivariable regression. See Appendix 1 for details of variable availability and wording between CCHS cycles.

Reviewer Comment: Inclusion and Exclusion Criteria not mentioned.
Author Response: Inclusion and exclusion criteria was added on the CCHS. This now reads: The CCHS covers the population 12 years of age and over who live in Canada. Excluded from the CCHS are institutionalized residents, full-time members of the Canadian Armed Forces, ~~residents of certain remote regions,~~ or people living on reserves/other Indigenous settlements in the provinces. Altogether, these exclusions represent less than 3% of the target population.

Reviewer Comment: Which type of logistic regression were used? Why? How?
Author Response: We clarified that we used survey-weighted multivariable logistic regression fitted separately for each provider and now reads: To identify factors associated with provider-specific mental health service we fit four separate, survey-weighted multivariable logistic regression models using the 2019-2020 cycle of the data for each provider type (family doctor, psychiatrist, psychologist, and social worker).

Reviewer Comment: Univariate and multivariate logistic regression analysis not performed, why?
Author Response: We did not perform univariate screening instead each provider-specific outcome was modeled with the prespecified multivariable set to avoid data-driven selection, preserve comparability across providers, and prevent exclusion of non-significant yet confounding covariates. Additionally, because key sociodemographic and health factors are correlated and rarely operate in isolation (e.g., income, education, racialization), multivariable models are required to estimate adjusted associations. This approach aligns with methodological guidance that discourages univariable screening in favor of theory-driven adjustment.

Reviewer Comment: Explain the logistic regression analysis in detail in the statistical analysis.
Author Response: We have added more detail into this statistical analysis and now reads as: An exploratory descriptive analysis was conducted to examine trends in mental health service use across provider types over time (2007-2020). To identify factors associated with provider-specific mental health service we fit four separate, survey-weighted multivariable logistic regression models using the 2019-2020 cycle of the data for each provider type (family doctor, psychiatrist, psychologist, and social worker). The models access the relationship between the use of a specific provider adjusting for all other covariates. Each model included the same a priori covariates listed above to keep models comparable. Outcomes were binary (any past-12-month contact with the provider: yes/no). We modeled providers separately because respondents may see multiple providers and access differs for various reasons including pathways and financing. A single multinomial model would also impose inappropriate mutual exclusivity. Two-tailed tests with a threshold of $\alpha = 0.05$ were used for all statistical tests. All analyses were conducted using R statistical software and survey weights were applied to account for the complex sampling design of the CCHS and to be representative of the Canadian population.

Reviewer Comment: Presenting the results in the table 1 not enough indicated; Revise the comments for logistic regression.
Author Response: Thank you for this suggestion. We revised the Results to add clear, in-text interpretation of Table 1. We now summarize the key statistically significant findings in four short paragraphs organized by provider and include a brief non-findings paragraph to for simplicity. We also strengthened the Table 1 caption and notes.

Reviewer Comment: Discussion: Looks good.
Author Response: Thank you

Reviewer Comment: Conclusion: Looks good.
Author Response: Thank you

Reviewer Comment: References: Looks good.

Powered by Editorial Manager® and ProduXion Manager® from Aries Systems Corporation

Author Response: Thank you

Reviewer Comment: Figures and tables: Figures looks good.
Author Response: Thank you

Reviewer Comment:  Some more issues should be considered necessary for publication: Suggestions for future studies also be mentioned.
Author Response: Thank you for this suggestion. We have a added a section on future directions for research that reads: To build on these findings, future research should focus on using longitudinal or linked survey administrative data to map sequences of care, switching between providers, and duration/intensity of treatment. Similarly, methods that focus on concurrent mental health service provider use would be valuable allowing for the use of multinomial models and to better understanding how individuals are accessing the mental health care system. Equity should be a focus allowing exploration on intersectional effects across gender, age, income, education, racialization, immigration, Indigenous identity, and urbanicity. Likewise, further exploration is needed on needs and barriers including coverage, out-of-pocket costs, wait times, language, and providers availability. Lastly, qualitative research with equity seeking groups can illuminate preferences, experiences, and structural barriers that quantitative data alone may miss.

Reviewer Comment: Please provide at least two related strengths for MS.
Author Response: Thank you for this suggestion. We have added a strength section that reads: This study has several strengths. First, it leverages the Canadian Community Health Survey across seven cycles (2007–2020), yielding nationally representative estimates and trends, with Statistics Canada’s sampling procedures and survey weights enhancing generalizability. Similarly, we use survey-weighted and fit provider-specific models (family doctor, psychiatrist, psychologist, social worker) allowing for a nuanced view of provider specific mental health care. Furthermore, the multivariable models are a priori and equity-focused, adjusting for correlated sociodemographic and health factors to explore variables related to policy.

Reviewer #2

Reviewer Comment: There are numerous grammatical errors and awkward sentence constructions. This research will addresses key knowledge gaps... should be corrected to This research addresses key knowledge gaps....Consider thorough proofreading or language editing to improve readability and professionalism.

Author Response: Thank you for reviewing this manuscript. We have gone through the manuscript for proofreading to improve readability.

Reviewer Comment: The abstract is too long and includes excessive methodological detail. Condense the abstract and emphasize key findings and implications more succinctly.

Author Response: We have edited to abstract to be more concise and focus on key findings.

Reviewer Comment: You mention that logistic regressions were only done for 2019–2020 due to differences in variable measurement across survey cycles. However, you do not provide details or references showing which variables changed and how. Add an appendix table comparing variable definitions across cycles.

Author Response: Thank you for this suggestion. We have added Appendix 1: Variable Definitions and Availability Across CCHS Cycles (2007–2020), which lists each construct and the availability of the variable in each cycle and any changes to the variable. Reasons for conducting logistic regressions on the 2019-2020 cycle include visible minority only being available in the 2019-2020 cycle, indigenous identity first appearing n the 2015-2016 cycle, and household income being measured differently prior to 2013. We also added a sentence in Methods directing readers to Appendix 1

for further details on the variables and the CCHS cycles.

Reviewer Comment: The exclusion of provider overlap in the logistic models is a serious limitation. Many people access multiple providers. Discuss this more explicitly, and consider sensitivity analyses or multinomial models if feasible.

Author Response: We agree that the concurrent use is important. We now state explicitly in the results and limitations that outcomes were modeled separately as any contact per provider. For example, in the results we have added: Because outcomes were modeled separately the estimates below should be interpreted as provider-specific associations. And in the limitation we have added: Likewise, given we did not model concurrent use across providers the results do not capture substitution, sequencing, or intensity of multi-provider care. Additionally, the CCHS lacks questions on visit timing, referral pathways, and number of contacts, limiting our ability to characterize multi-provider pathways. Furthermore, we did not have measures of symptom severity, diagnosis, functional impairment, prior treatment, local provider supply, wait times, out-of-pocket costs, or insurance and EAP coverage. We also lacked information on language, travel time, and referral practices. The lack of these factors does not provide a full picture of mental health service use and the omission of these factors could confound associations if they correlate with both with both sociodemographic characteristics and provider use.Because of this our findings should be interpreted as adjusted associations rather than causal effects. Future studies with linked longitudinal data and methods that explicitly accommodate overlap is needed. We considered the suggested sensitivity analyses and multinomial models but did not implement them for methodological and practical reasons. For example, multinomial models required many overlap categories and with small cell counts this will lead to instability under the complex survey design.

Reviewer Comment: Table 1 is dense and hard to interpret. Consider moving odds ratios and confidence intervals to supplemental material and highlighting only significant findings in the main text. You could benefit from a heatmap-style table or bolding to visually distinguish significant results.

Author Response: Thank you for this helpful suggestion. We retained the full numeric Table 1 to preserve exact effect sizes and confidence intervals. To improve readability, we bolded all statistically significant AORs additionally we have updated the results section to focus on each model to ease with readability. These changes to Table 1 make it easier to read while maintaining transparency.

Reviewer Comment: The manuscript references Figures 1 and 2, but they are not embedded or summarized effectively in the results. Embed or summarize the main trends and include captions that explain anomalies (2017 drop).

Author Response: Thank you for this suggestion we have edited the text to better summarize the main trends. This section now reads for figure 1: Between 2007 and 2020, family doctors consistently accounted for the highest proportion of mental health service use in Canada in every cycle with over half of service users responding to having accessed a family doctor for mental health care (See Figure 1). Across all cycles, the rank order of providers was stable and the pattern over the years are relatively stable, with only modest year-to-year variation The proportion of people who accessed care through psychologists and social workers showed a gradual increase over time, while the proportion of visits to psychiatrists remained relatively unchanged over time. A one-year divergence appears in 2017, with a temporary spike for psychologist and dips slightly for the other providers but by the next cycle (2019) the pattern returns with psychologist and social workers converging at similar levels. We interpret 2017 as single cycle fluctuation rather than a break in trend. Estimates are survey-weighted and reflect that provider contacts are not mutually exclusive
And for figure 2 the description now reads: When examining weighted estimates of the number of Canadians accessing care, similar trends were observed (See Figure 2). The total number of individuals seeing family doctors for mental health reasons far exceeded those accessing psychologists, psychiatrists, or social workers. However, all provider types showed increases in absolute numbers over time reflecting increased mental service use overtime. Notably, a temporary drop in service use was observed in

Powered by Editorial Manager® and ProduXion Manager® from Aries Systems Corporation

2017 with a rebound by 2019 across all provider types, potentially reflecting data collection limitations in that survey cycle. Given each cycle of the CCHS has different weight calibrations and sample allocations, we believe this is a cycle level fluctuation rather than a structural change to mental health care use.

Reviewer Comment: The discussion covers many findings but often lacks depth in interpretation. Provide more nuance on why certain sociodemographic factors (e.g., education, income) are predictive of use for certain providers. Integrate more Canadian-specific policy recommendations and barriers (wait times, geographic access, EAP programs).

Author Response: Thank you for this suggestion we have added throughout to the discussion section. For example we have added more on age as predictor: Furthermore, these age patterns likely also reflect differences in care continuity and navigation. Older adults have stronger ties to a usual family doctor and more multimorbidity which makes primary care a natural entry point for mental and physical concerns. Younger adults in contrast more often have access to private insurance and Employee Assistance Programs (EAP) for counselling which may lower the threshold to see psychologist and social workers.
Costs: Specifically, in Canada psychologist and social workers are not universally covered and access often depends on employers benefits, EAPs, or private insurance which commonly cap the number of sessions. By contrast, visits to family doctors and psychiatrist are publicly insured which may explain a shift towards these providers among lower income groups.
Geographic disparities: Furthermore, these disparities are also shaped by geography and provider supply. For example, mental health care providers are often concentrated in large urban centre resulting in longer travel times and indirect costs for rural communities to accessing care.

Canadian-specific policy recommendations: Several actions could improve equitable access to mental health services in Canada. First, expanding publicly funded, evidence-based psychotherapy would reduce the role of financial barriers. Second, integrating mental health care providers into primary care teams by streamlining referrals, provided shared care can result in shortened wait times and improved continuity in care. Equity can be further strengthened through Indigenous-led services, cultural-safety standards, language interpretation, and navigation supports for newcomers and racialized communities. Furthermore, private insurance and EAPs need to be modernized to ensure adequate session coverage. Lastly, further investment needs to be made in community-based mental health care including walk-in counselling, youth hubs, peer and Indigenous led programs and system navigation would expand timely service and reduce avoidable reliance on emergency and specialist services.

Reviewer Comment: The limitations section is too brief. Elaborate on limitations of self-report data, cross-sectional design, and unobserved confounders (e.g., provider availability, wait times, severity of need).

Author Response: Thank you for this suggestion. We have updated the limitations section, and it now reads as: This study has several limitations. First, mental health service use was based on self-report and may be subject to recall or reporting biases. Second, only service use within the past year was captured without accounting for frequency, duration, or perceived adequacy of care. Third, the regression models only used cross-sectional data from 2019–2020. This is due to an inability to combine data from different cycles together as predictor variables were measured differently across various cycles of the survey. This means that logistic regression results are not able to describe longer-term patterns. Additionally, service use was measured separately for each provider type, without information on concurrent or integrated care. Likewise, given we did not model concurrent use across providers the results do not capture substitution, sequencing, or intensity of multi-provider care. Additionally, the CCHS lacks questions on visit timing, referral pathways, and number of contacts, limiting our ability to characterize multi-provider pathways. Furthermore, we did not have measures of symptom severity, diagnosis, functional impairment, prior treatment, local provider supply, wait times, out-of-pocket costs, or insurance and EAP coverage. We also lacked information on language, travel time, and referral practices. The lack of these

| | |
|---|---|
| | factors does not provide a full picture of mental health service use and the omission of these factors could confound associations if they correlate with both with both sociodemographic characteristics and provider use. Because of this our findings should be interpreted as adjusted associations rather than causal effects. Future studies with linked longitudinal data and methods that explicitly accommodate overlap is needed.<br><br>Reviewer Comment: While you mention Statistics Canada data, the Data Availability Statement is vague. Include exact dataset names, links to access procedures, and R code availability (if possible).<br><br>Author Response: We have revised the data availability statement to include information and new reads:<br><br>Data Access The data that support the findings of this study are provided by Statistics Canada microdata files. This study used the Canadian Community Health Survey (CCHS) Public Use Microdata Files (PUMF) for the following cycles: 2007–2008, 2009–2010, 2011–2012, 2013–2014, 2015–2016, 2017–2018, and 2019–2020.<br>The dataset names are as follows:<br>1.Canadian Community Health Survey, 2007-2008: Annual Component<br>2.Canadian Community Health Survey, 2009-2010: Annual Component<br>3.Canadian Community Health Survey, 2011-2012: Annual Component<br>4.Canadian Community Health Survey, 2013-2014: Annual Component<br>5.Canadian Community Health Survey, 2015-2016: Annual Component<br>6.Canadian Community Health Survey, 2017-2018: Annual Component<br>7.Canadian Community Health Survey, 2019-2020: Annual Component<br><br>Files were accessed through ODESI via the University of Toronto. The R scripts used for data preparation, survey-weighted analyses, and figure generation are available from the corresponding author upon reasonable request for research purposes.<br><br>Reviewer Comment: Include OR values in the abstract for the most important findings. Improve consistency in terminology — e.g., switch between visible minority and non-visible minority inconsistently.<br><br>Author Response: Thank you for this suggestion. We have edited to the abstract to include OR values and have gone through the manuscript for consistency in terminology. |
| **Additional Information:** | |
| **Question** | **Response** |
| **Financial Disclosure**<br><br>Enter a financial disclosure statement that describes the sources of funding for the work included in this submission. Review the submission guidelines for detailed requirements. View published research articles from *PLOS ONE* for specific examples.<br><br>This statement is required for submission and **will appear in the published article** if the submission is accepted. Please make sure it is accurate. | The author(s) received no specific funding for this work. |

| | |
|---|---|
| **Funded studies**<br>Enter a statement with the following details:<br>• Initials of the authors who received each award<br>• Grant numbers awarded to each author<br>• The full name of each funder<br>• URL of each funder website<br>• Did the sponsors or funders play any role in the study design, data collection and analysis, decision to publish, or preparation of the manuscript?<br><br>Did you receive funding for this work? | |
| **Competing Interests**<br><br>Use the instructions below to enter a competing interest statement for this submission. On behalf of all authors, disclose any competing interests that could be perceived to bias this work—acknowledging all financial support and any other relevant financial or non-financial competing interests.<br><br>This statement is required for submission and **will appear in the published article** if the submission is accepted. Please make sure it is accurate and that any funding sources listed in your Funding Information later in the submission form are also declared in your Financial Disclosure statement.<br><br>View published research articles from *PLOS ONE* for specific examples. | The authors have declared that no competing interests exist. |

*Powered by Editorial Manager® and ProduXion Manager® from Aries Systems Corporation*

| | |
|---|---|
| **NO authors have competing interests**<br><br>Enter: *The authors have declared that no competing interests exist.*<br><br>**Authors with competing interests**<br><br>Enter competing interest details beginning with this statement:<br><br>*I have read the journal's policy and the authors of this manuscript have the following competing interests: [insert competing interests here]*<br><br>* typeset | |
| **Ethics Statement**<br><br>Enter an ethics statement for this submission. This statement is required if the study involved:<br><br>• Human participants<br>• Human specimens or tissue<br>• Vertebrate animals or cephalopods<br>• Vertebrate embryos or tissues<br>• Field research<br><br>Write "N/A" if the submission does not require an ethics statement.<br><br>General guidance is provided below. Consult the submission guidelines for detailed instructions. **Make sure that all information entered here is included in the Methods section of the manuscript.** | This study used retrospective survey data from the Canadian Community Health Survey Public Use Microdata Files (PUMFs), accessed for research purposes in January 2025. These data are collected and publicly released by Statistics Canada, which obtains informed consent from participants at the time of original data collection. The PUMFs are fully anonymized prior to release and do not contain any identifying information. As a result, the authors did not have access to any information that could identify individual participants during or after data collection, and additional institutional ethics approval was not required for this secondary analysis. |

Powered by Editorial Manager® and ProduXion Manager® from Aries Systems Corporation

| | |
|---|---|
| **Format for specific study types**<br><br>**Human Subject Research (involving human participants and/or tissue)**<br>• Give the name of the institutional review board or ethics committee that approved the study<br>• Include the approval number and/or a statement indicating approval of this research<br>• Indicate the form of consent obtained (written/oral) or the reason that consent was not obtained (e.g. the data were analyzed anonymously)<br><br>**Animal Research (involving vertebrate animals, embryos or tissues)**<br>• Provide the name of the Institutional Animal Care and Use Committee (IACUC) or other relevant ethics board that reviewed the study protocol, and indicate whether they approved this research or granted a formal waiver of ethical approval<br>• Include an approval number if one was obtained<br>• If the study involved *non-human primates*, add *additional details* about animal welfare and steps taken to ameliorate suffering<br>• If anesthesia, euthanasia, or any kind of animal sacrifice is part of the study, include briefly which substances and/or methods were applied<br><br>**Field Research**<br><br>Include the following details if this study involves the collection of plant, animal, or other materials from a natural setting:<br>• Field permit number<br>• Name of the institution or relevant body that granted permission | |
| **Data Availability**<br><br>Authors are required to make all data underlying the findings described fully available, without restriction, and from the time of publication. PLOS allows rare exceptions to address legal and ethical concerns. See the PLOS Data Policy and FAQ for detailed information. | No - some restrictions will apply |

Powered by Editorial Manager® and ProduXion Manager® from Aries Systems Corporation

| | |
|---|---|
| A Data Availability Statement describing where the data can be found is required at submission. Your answers to this question constitute the Data Availability Statement and **will be published in the article**, if accepted.<br><br>**Important:** Stating 'data available on request from the author' is not sufficient. If your data are only available upon request, select 'No' for the first question and explain your exceptional situation in the text box.<br><br>Do the authors confirm that all data underlying the findings described in their manuscript are fully available without restriction? | |
| **Describe where the data may be found in full sentences. If you are copying our sample text, replace any instances of XXX with the appropriate details.**<br><br>• If the data are **held or will be held in a public repository**, include URLs, accession numbers or DOIs. If this information will only be available after acceptance, indicate this by ticking the box below. For example: *All XXX files are available from the XXX database (accession number(s) XXX, XXX.*).<br>• If the data are all contained **within the manuscript and/or Supporting Information files**, enter the following: *All relevant data are within the manuscript and its Supporting Information files.*<br>• If neither of these applies but you are able to provide **details of access elsewhere**, with or without limitations, please do so. For example:<br><br>*Data cannot be shared publicly because of [XXX]. Data are available from the XXX Institutional Data Access / Ethics Committee (contact via XXX) for researchers who meet the criteria for access to confidential data.*<br><br>*The data underlying the results presented in the study are available from (include the name of the third party* | The data used in this study are third-party data owned by Statistics Canada and cannot be shared by the authors. We analyzed the Canadian Community Health Survey (CCHS), Annual Component – Public Use Microdata Files (PUMF) for cycles 2007–2008, 2009–2010, 2011–2012, 2013–2014, 2015–2016, 2017–2018, and 2019–2020. Files were obtained via Odesi (Open Data Documentation, Extraction Service and Infrastructure) through the University of Toronto Libraries under the Data Liberation Initiative (DLI) license. Researchers at DLI-participating institutions can access the same PUMFs through their library's Odesi/Dataverse portal (https://odesi.ca). Others may obtain CCHS PUMFs from Statistics Canada's data portal (https://www150.statcan.gc.ca) or apply for access to the confidential master files via the Canadian Research Data Centre Network (CRDCN) (https://crdcn.ca), subject to approval and confidentiality requirements. The authors had no special access privileges that others would not have. |

Powered by Editorial Manager® and ProduXion Manager® from Aries Systems Corporation

| | |
|---|---|
| *and contact information or URL).*<br>• This text is appropriate if the data are owned by a third party and authors do not have permission to share the data.<br><br>* typeset | |
| Additional data availability information: | |

Manuscript Click here to access/download;Manuscript;Manuscript.docx

**Sociodemographic and Mental Health Predictors of Mental Health Service Use Across Provider Types**

Nelson Pang, MSW,[1] Jessie Yeung, MSc[2]

[1] Factor-Inwentash Faculty of Social Work, University of Toronto, Toronto, Ontario, Canada

[2] Department of Statistical Sciences, University of Toronto, Toronto, Ontario, Canada

**Author Note**

Correspondence concerning this article should be addressed to Nelson Pang, Factor-Inwentash Faculty of Social Work, University of Toronto, 246 Bloor St W, Toronto, Ontario, Canada, M5S 1V4. Email: nelson.pang@mail.utoronto.ca

**Abstract**

*Objective:* To examine trends in mental health service use across four provider types (family doctors, psychiatrists, psychologists, and social workers) and identify sociodemographic predictors of provider-specific access in Canada.

*Methods:* This study analyzes seven cycles (2007–2020) of the Canadian Community Health Survey, a nationally representative cross-sectional survey. Trends over time were examined using weighted proportions and counts of service users. Weighted multivariable logistic regression models were applied to the 2019–2020 cycle to assess associations between sociodemographic factors and provider-specific service use.

*Results:* Family doctors were consistently the most accessed providers for mental health concerns, followed by psychologists and social workers, with psychiatrists being least accessed. Psychologist and social worker use increased between 2017 and 2019. In the adjusted regression models (2019–2020), women had higher odds of using family doctors (AOR = 1.21, 95% CI: 1.05–1.39) and social workers (1.19, 1.02–1.40) and lower odds of psychiatrists (AOR = 0.66, 95% CI:0.55–0.79) than men. Adults 65+ had greater odds of family-doctor use (AOR = 4.82, 95% CI: 3.59–6.47) and lower odds of psychologist (AOR = 0.33, 95% CI: 0.24–0.45) and social-worker use (AOR = 0.21, 95% CI:0.16–0.29) than ages 12–17. Post-secondary education (vs less than secondary school) was associated with higher psychologist use (AOR = 1.83, 95% CI 1.12–2.98). Higher income (≥$80,000 vs <$20,000) was associated with lower psychiatrist use (AOR = 0.66, 95% CI 0.50–0.86). Non-Indigenous respondents more often used psychologists (AOR = 1.58, 95% CI: 1.13–2.23), and respondents who are not a visible minority more often used family doctors (AOR = 1.37, 95% CI:1.06–1.77).

*Conclusion:* This study reveals a stratified mental health care system in Canada, where sociodemographic factors shape who accesses which providers. While primary care dominates, growth in psychologist and social worker use suggests shifting patterns of engagement. Findings underscore the need for policies that address financial and structural barriers, promote equitable access, and expand coverage for community-based mental health providers.

**Keywords:** Mental Health Care Access, Sociodemographic disparities, Health Care Access, Psychiatric care, Health Service Trends

## Background

Mental health is a significant public health concern in Canada, with approximately 1 in 5 Canadians experiencing a mental illness every year.[1] Despite this access to mental health care is limited with studies estimating that only around 40–60% of people with a mental health problem seek professional assistance.[2,3] In 2018, an estimated 5.3 million Canadians reported needing help for their mental health in the previous year highlighting the widespread demand for mental health support across the Canadian population.[4] Alarmingly, nearly half of these individuals had their needs only partially met (1.2 million) or completely unmet (1.1 million), suggesting substantial gaps in service delivery for mental health. [4]

In Canada, mental health care is provided by a diverse range of professionals, including psychiatrists, psychologists, social workers, nurses, and physicians.[3] These different providers differ not only in their training and scope of practice but also in funding and accessibility.[5] For example, family physicians and psychiatrists are typically publicly funded and can be billed directly to provincial and territorial health insurance plans whereas services provided by psychologists and social workers may require out-of-pocket payment or private insurance coverage. [3] These differences in cost of services may result in financial barriers for individuals seeking care. Furthermore, these differences have implications on who receives care, what kind of care is delivered, and how barriers to access may differ across populations. In recent years, the mental health service landscape in Canada has become increasingly complex with expanding roles in the mental health care for non-physician providers such as psychologists and social workers.[5] This change reflects both efforts to meet growing demand for mental health care, shifts toward evidence-based care, and interprofessional models of service delivery.[6,7]

Previous research has shown that mental health care utilization is shaped by an interplay of need, enabling factors and predisposing factors, such as symptom severity, income, gender, education, cultural background, wait time, and costs.[8-12] Despite Canada having universal health insurance, disparities and inequities are still associated with the underutilization of mental health care services.[8,11,13,14] For example, previous research in Canada has found that individuals with higher education are more likely to utilize mental health services than those in less educated groups.[11,13] Likewise, previous research has found income-based inequity in access to mental health services.[8,14] Understanding which populations access specific types of providers is critical for informing mental health care and equity-focused policies. For example, investments in community-based providers such as psychologists and social workers may reduce reliance on primary care for mental health, improve continuity of care, and expand culturally appropriate service options.[15-17]

A limitation in this research however is that most studies aggregate all types of mental health providers into one variable limiting the examination of differences in who accesses which services and under which circumstances. As a result, we lack nuanced understanding of how sociodemographic factors shape engagement with specific provider types, such as psychologists or social workers, whose roles and funding structures differ significantly from psychiatrists and family doctors. Furthermore, few studies have examined national level trends over time in provider specific mental health service use limiting our ability to assess how patterns have shifted in response to evolving service landscapes, policy changes, or public attitudes towards mental health. To address these gaps, this study focuses on examining how sociodemographic and mental health characteristics are associated with mental health service use across four provider types (family doctors, psychiatrists, psychologists, and social workers) and trends in

their use over time in Canada. By disaggregating service use by provider type, this research aims to uncover distinct access patterns across sociodemographic groups and inform efforts to promote equity in mental health care.

## Methods

### *Study Design and Data Source*

We conducted a cross-sectional, population-based analysis using seven cycles (2007-2020) of the Canadian Community Health Survey (CCHS) public-use microdata. The CCHS is a nationally representative cross-sectional survey administered by Statistics Canada. The CCHS is a self-reported survey on health, healthcare utilization, and health determinants of the Canadian population aged 12 and above. The CCHS covers the population 12 years of age and over who live in Canada. Excluded from the CCHS are institutionalized residents, full-time members of the Canadian Armed Forces, residents of certain remote regions, or people living on reserves/other Indigenous settlements in the provinces. Altogether, these exclusions represent less than 3% of the target population. Sample sizes varied across cycles, and all analyses applied survey weights provided by Statistics Canada to ensure representativeness of the Canadian population. This study summarized trends of provider specific mental health access across 2007–2020 and provider specific logistic regressions were restricted to 2019–2020 because it was the most recent cycle and contained key predictors specified a priori for multivariable regression. See Appendix 1 for details of variable availability and wording between CCHS cycles.

### *Ethics, Data Access, and Participant Confidentiality*

This study used retrospective survey data from the CCHS Public Use Microdata Files (PUMFs), accessed for research purposes in January 2025. These data are collected and publicly

released by Statistics Canada, which obtains informed consent from participants at the time of original data collection. The PUMFs are fully anonymized prior to release and do not contain any identifying information. As a result, the authors did not have access to any information that could identify individual participants during or after data collection, and additional institutional ethics approval was not required for this secondary analysis.

*Outcome variables*

The data was subsetted to only include respondents who have consulted a mental health professional in the past 12-months (n=13,580/weighted n= 4,679,132.44). The primary outcome were binary indicators of if respondents reported having seen or talked to a specific health professional based on the question *"In the past 12 months have you seen or talked to a health professional about your emotional or mental health?"*. The analysis focused on the four most common providers for mental health care (family doctor, psychiatrist, psychologist, and social work). Each provider was coded as a separate variable as respondents could see more than one provider.

*Predictor variables*

A variety of sociodemographic and mental health characteristics were included as predictors in the model. Sociodemographic characteristics included sex, age, household income, being a visible minority, education, immigration status, and Aboriginal identity. Mental health characteristics included perceived health and perceived mental health.

*Statistical Analysis*

An exploratory descriptive analysis was conducted to examine trends in mental health service use across provider types over time (2007-2020). To identify factors associated with provider-specific mental health service we fit four separate, survey-weighted multivariable

logistic regression models using the 2019-2020 cycle of the data for each provider type (family doctor, psychiatrist, psychologist, and social worker). The models access the relationship between the use of a specific provider adjusting for all other covariates. Each model included the same a priori covariates listed above to keep models comparable. Outcomes were binary (any past-12-month contact with the provider: yes/no). We modeled providers separately because respondents may see multiple providers and access differs for various reasons including pathways and financing. A single multinomial model would also impose inappropriate mutual exclusivity. Two-tailed tests with a threshold of $\alpha = 0.05$ were used for all statistical tests. All analyses were conducted using R statistical software and survey weights were applied to account for the complex sampling design of the CCHS and to be representative of the Canadian population.

**Results**

Between 2007 and 2020, family doctors consistently accounted for the highest proportion of mental health service use in Canada in every cycle with over half of service users responding to having accessed a family doctor for mental health care (See Figure 1). Across all cycles, the rank order of providers was stable and the pattern over the years are relatively stable, with only modest year-to-year variation The proportion of people who accessed care through psychologists and social workers showed a gradual increase over time, while the proportion of visits to psychiatrists remained relatively unchanged over time. A one-year divergence appears in 2017, with a temporary spike for psychologist and dips slightly for the other providers but by the next cycle (2019) the pattern returns with psychologist and social workers converging at similar levels. We interpret 2017 as single cycle fluctuation rather than a break in trend. Estimates are survey-weighted and reflect that provider contacts are not mutually exclusive.

*Figure 1. Past-12-month contact for mental health by provider, CCHS 2007–2019 (survey-weighted proportion)*

When examining weighted estimates of the number of Canadians accessing care, similar trends were observed (See Figure 2). The total number of individuals seeing family doctors for mental health reasons far exceeded those accessing psychologists, psychiatrists, or social workers. However, all provider types showed increases in absolute numbers over time reflecting increased mental service use overtime. Notably, a temporary drop in service use was observed in 2017 with a rebound by 2019 across all provider types, potentially reflecting data collection limitations in that survey cycle. Given each cycle of the CCHS has different weight calibrations and sample allocations, we believe this is a cycle level fluctuation rather than a structural change to mental health care use.

*Figure 2. Weighted number of Canadians reporting past-12-month mental-health contact by provider, CCHS 2007–2019*

Adjusted associations between sociodemographic and mental health characteristics and provider-specific mental health service use are presented in Table 1. Because outcomes were modeled separately the estimates below should be interpreted as provider-specific associations.

Table 1. Adjusted Associations between Sociodemographic Variables and Mental Health Provider Specific Mental Health Service Use

| | **Social Work** | **Family Doctor** | **Psychiatry** | **Psychologist** |
|---|---|---|---|---|

| | AOR | 95% CI | p-value | AOR | 95% CI | p-value | AOR | 95% CI | p-value | AOR | 95% CI | p-value |
|---|---|---|---|---|---|---|---|---|---|---|---|---|
| **Sex**<br>(Ref: Men) | | | | | | | | | | | | |
| Women | **1.19** | **1.02, 1.4** | **0.028** | **1.21** | **1.05, 1.39** | **0.007** | **0.66** | **0.55, 0.79** | **<.001** | 0.98 | 0.84, 1.14 | 0.760 |
| **Age**<br>(Ref: 12-17) | | | | | | | | | | | | |
| 18-34 | **0.49** | **0.38, 0.63** | **<.001** | **2.97** | **2.26, 3.91** | **<.001** | 0.82 | 0.55, 1.22 | 0.325 | 0.78 | 0.59, 1.03 | 0.084 |
| 35-49 | **0.38** | **0.3, 0.49** | **<.001** | **3.74** | **2.86, 4.9** | **<.001** | 0.62 | 0.42, 0.91 | 0.014 | 0.93 | 0.71, 1.22 | 0.606 |
| 50-64 | **0.25** | **0.18, 0.33** | **<.001** | **3.98** | **3.01, 5.26** | **<.001** | 0.83 | 0.56, 1.22 | **0.343** | **0.69** | **0.52, 0.92** | **0.011** |
| 65+ | **0.21** | **0.16, 0.29** | **<.001** | **4.82** | **3.59, 6.47** | **<.001** | 0.96 | 0.64, 1.44 | **0.839** | **0.33** | **0.24, 0.45** | **<.001** |
| **Household Income**<br>(Ref: No income or less than $20,000) | | | | | | | | | | | | |
| $20,000 to $39,999 | 0.95 | 0.69, 1.31 | 0.759 | **1.37** | **1.04, 1.81** | **0.027** | 0.82 | 0.6, 1.12 | 0.203 | 0.87 | 0.62, 1.23 | 0.444 |
| $40,000 to $59,999 | 0.89 | 0.65, 1.22 | 0.478 | **1.53** | **1.15, 2.03** | **0.004** | **0.7** | **0.5, 0.97** | **0.031** | 0.93 | 0.67, 1.31 | 0.694 |
| $60,000 to $79,999 | 0.82 | 0.59, 1.14 | 0.234 | 1.32 | 0.98, 1.77 | 0.070 | **0.6** | **0.42, 0.85** | **0.004** | 1.14 | 0.77, 1.68 | 0.507 |
| $80,000 or more | 0.90 | 0.68, 1.19 | 0.472 | **1.41** | **1.11, 1.8** | **0.006** | **0.66** | **0.5, 0.86** | **0.002** | 1.09 | 0.82, 1.46 | 0.546 |
| **Visible Minority**<br>(Ref: Visible Minority) | | | | | | | | | | | | |
| Not a visible minority | 1.05 | 0.79, 1.41 | 0.717 | **1.37** | **1.06, 1.77** | **0.017** | 1.23 | 0.85, 1.8 | 0.270 | 1.2 | 0.88, 1.63 | 0.245 |
| **Highest level of education in household**<br>(Ref: Less than secondary school graduation) | | | | | | | | | | | | |

| | | | | | | | | | | | | |
|---|---|---|---|---|---|---|---|---|---|---|---|---|
| Secondary school graduation, no post-secondary education | 0.96 | 0.62, 1.49 | 0.862 | 0.76 | 0.54, 1.08 | 0.128 | 1.12 | 0.73, 1.72 | 0.598 | 1.28 | 0.74, 2.24 | 0.378 |
| Post-secondary certificate/diploma / university degree | 1.08 | 0.72, 1.62 | 0.706 | **0.61** | **0.45, 0.83** | **0.002** | 1.4 | 0.95, 2.08 | 0.093 | **1.83** | **1.12, 2.98** | **0.016** |
| **Aboriginal identity - First nations / Metis / Inuk (Inuit)** (Ref: Yes) | | | | | | | | | | | | |
| No | 0.84 | 0.63, 1.13 | 0.262 | 1.12 | 0.84, 1.49 | 0.423 | 1.1 | 0.75, 1.61 | 0.636 | **1.58** | **1.13, 2.23** | **0.008** |
| **Immigrant** (Ref: Landed immigrant / non-permanent resident) | | | | | | | | | | | | |
| Non-immigrant (Canadian born) | 1 | 0.77, 1.29 | 0.990 | 1 | 0.8, 1.26 | 0.970 | 1.27 | 0.95, 1.7 | 0.111 | 0.93 | 0.71, 1.21 | 0.567 |
| **Perceived Health** (Ref: Poor) | | | | | | | | | | | | |
| Fair | 0.74 | 0.53, 1.04 | 0.086 | 0.9 | 0.66, 1.23 | 0.509 | **0.68** | **0.48, 0.94** | **0.022** | 1.22 | 0.85, 1.76 | 0.284 |
| Good | 0.73 | 0.53, 1 | 0.051 | 1.02 | 0.76, 1.37 | 0.883 | **0.56** | **0.41, 0.77** | **<.001** | 1.24 | 0.89, 1.72 | 0.200 |
| Very good | 0.73 | 0.52, 1.02 | 0.062 | 0.91 | 0.67, 1.24 | 0.553 | **0.39** | **0.27, 0.55** | **<.001** | 1.33 | 0.94, 1.88 | 0.102 |
| Excellent | 0.72 | 0.49, 1.07 | 0.103 | 0.78 | 0.56, 1.11 | 0.165 | **0.34** | **0.22, 0.52** | **<.001** | **1.47** | **1.01, 2.15** | **0.045** |
| **Perceived Mental Health** (Ref: Poor) | | | | | | | | | | | | |
| Fair | 0.79 | 0.59, 1.05 | 0.101 | 0.91 | 0.68, 1.23 | 0.553 | **0.52** | **0.38, 0.7** | **<.001** | 0.93 | 0.66, 1.3 | 0.662 |
| Good | **0.65** | **0.49, 0.87** | **0.004** | 0.74 | **0.55, 1** | **0.050** | **0.33** | **0.24, 0.44** | **<.001** | 0.92 | 0.66, 1.28 | 0.618 |
| Very good | **0.63** | **0.46, 0.86** | **0.004** | 0.57 | **0.42, 0.78** | **<.001** | **0.28** | **0.2, 0.41** | **<.001** | 0.9 | 0.63, 1.28 | 0.559 |
| Excellent | **0.64** | **0.44, 0.94** | **0.024** | 0.58 | **0.41, 0.84** | **0.003** | **0.24** | **0.16, 0.37** | **<.001** | 0.84 | 0.56, 1.26 | 0.400 |

| | | | | | | | | | | | | |
|---|---|---|---|---|---|---|---|---|---|---|---|---|
| (Intercept) | 1.77 | 0.9, 3.5 | 0.101 | **0.33** | **0.18, 0.61** | **<.001** | 1.02 | 0.47, 2.24 | 0.951 | **0.13** | **0.06, 0.28** | **<0.001** |
| Note. AOR: Adjusted Odds Ratio; CI: Confidence Interval; **Boldface** indicates statistical significance at the 0.05 level. | | | | | | | | | | | | |

Use of social workers was higher among women (AOR = 1.19, 95% CI: 1.02–1.40) and decreased with age across all groups relative to the 12–17 group (AOR [18–34] = 0.49, 95% CI: 0.38–0.63; AOR[35–49] = 0.38, 95% CI: 0.30–0.49; AOR [50–64] = 0.25, 95% CI: 0.18–0.33; AOR [65+] = 0.21, 95% CI: 0.16–0.29). Better perceived mental health was associated with lower odds compared to having poor perceived mental health (AOR [good] = 0.65, 95% CI: 0.49–0.87; AOR [very good] = 0.63, 95% CI: 0.46–0.86; AOR [excellent] = 0.64, 95% CI: 0.44–0.94).

Use of family doctors was higher among women than men (AOR = 1.21, 95% CI: 1.05–1.39) and increased sharply with age (vs. 12–17: AOR [18–34] = 2.97, 95% CI: 2.26–3.91; AOR[35–49] = 3.74, 95% CI: 2.86–4.90; AOR [50–64] = 3.98, 95% CI: 3.01–5.26; AOR [65+] = 4.82, 95% CI: 3.59–6.47). Higher household income was associated with higher odds of using a family doctor (vs. No income or less than $20,000. AOR[$40–59k]: 1.53, 95% CI:1.15–2.03; AOR[ ≥$80k]= 1.41, 95% CI: 1.11–1.80), whereas post-secondary education was associated with lower odds compared to those with less than secondary school graduation (AOR = 0.61, 95% CI: 0.45–0.83). Not being a visible minority was also associated with higher odds of seeing a family doctor (AOR: 1.37, 95% CI: 1.06–1.77). Better perceived mental health related to lower use compared to having poor perceived mental health (AOR [very good] = 0.57, 95% CI: 0.42–0.78; AOR [excellent] = 0.58, 95% CI: 0.41–0.84).

Odds of seeing a psychiatrist were lower for women than men (AOR = 0.66, 95% CI: 0.55–0.79) and lower for adults aged 35–49 relative to 12–17 (AOR = 0.62, 95% CI: 0.42–0.91). Higher income was associated with lower odds (vs. No income or less than $20,000. AOR [$40–59k] = 0.70, 95% CI: 0.50–0.97; AOR [$60–79k] = 0.60, 95% CI:0.42–0.85; APR [≥$80k] = 0.66, 95% CI: 0.50–0.86). Better perceived health and perceived mental health related to lower use (e.g., perceived health excellent vs poor perceived health. AOR = 0.34, 95% CI; 0.22–0.52; perceived mental health excellent. AOR = 0.24, 95% CI: 0.16–0.37).

Psychologist use declined at older ages (AOR [50–64] = 0.69, 95% CI:0.52–0.92; AOR [65+] = 0.33, 95% CI: 0.24–0.45) compared with the 12–17 age group. In contrast, post-secondary education was associated with higher odds (AOR =1.83, 95% CI: 1.12–2.98) compared to those with less than secondary school graduation. Being non-indigenous was associated with higher psychologist used compared to indigenous groups (AOR: 1.58, 95% CI: 1.13–2.23). Excellent perceived health was also associated with higher odds compared to those with poor perceived health (AOR: 1.47, 95% CI: 1.01–2.15).

Across providers, immigrant status was not significantly associated with mental health service use. Household income showed no association with psychologist or social worker use. Being a visible minority and Indigenous identity showed no associations overall, with two exceptions (respondents who were not a visible minority having higher odds of family-doctor use and non-Indigenous respondents having higher odds of psychologist use).

**Discussion**

This study examined national trends in mental health service use across four provider types (family doctors, psychiatrists, psychologists, and social workers) and identified

sociodemographic predictors to accessing each provider using a recent nationally representative survey. Results show that family doctors were the most common provider for mental health, followed by psychologists and social workers, while psychiatrists are accessed less frequently. These trends were generally stable over time, with increases in psychologist and social worker use observed between 2017 and 2019. Consistent with previous research, family doctors were found to be the most commonly accessed mental health care provider, reaffirming the central role of primary care in Canada's mental health system.[5,18] Given the high rates and numbers of people who seek mental health care from family doctors it is important that family doctors are trained in assessment and treatment of mental health.[19] Access to psychologists and social workers increased in recent years, particularly between 2017 and 2019, which may reflect changing public attitudes toward mental health, reduced stigma, and increased availability of services.[20-22]

Logistic regression analyses from the 2019–2020 CCHS cycle indicate significant differences in access to providers across different demographic groups. Women had higher odds of seeking support from family doctors and social workers compared to men, aligning with prior literature showing greater mental health service use among women.[23] Age emerged as a significant predictor of provider type with older adults more likely to seek care from family doctors and younger individuals more likely to access psychologists and social workers. This trend may reflect generational differences in mental health literacy, familiarity with provider types, and access pathways.[24,25] Additionally, mental health services delivered by psychologists and social workers are not universally covered by provincial health plans in Canada, and younger individuals may access these providers through post-secondary institutions, school-based programs, or employee insurance plans. The rise in mental health awareness among younger

generations, combined with greater comfort in discussing psychological issues, may also increase their likelihood of seeking specialized care.[26,27] In contrast, older adults may be more affected by stigma surrounding mental illness and thus prefer family doctors who can address mental and physical health simultaneously in a less stigmatizing setting.[25] Furthermore, these age patterns likely also reflect differences in care continuity and navigation. Older adults have stronger ties to a usual family doctor and more multimorbidity which makes primary care a natural entry point for mental and physical concerns. Younger adults in contrast more often have access to private insurance and Employee Assistance Programs (EAP) for counselling which may lower the threshold to see psychologist and social workers.

Income and education were associated with distinct patterns of provider use. Individuals with higher education were more likely to access psychologists, potentially reflecting the private-pay nature of these services and barriers related to insurance coverage. In contrast, individuals with lower income and education levels were more likely to see family doctors or social workers, who are often covered under public or community-based services. These patterns highlight ongoing structural inequities in access to mental health care, where the ability to pay often determines the type and intensity of support received.[28,29] These findings support the need for publicly funded access to a broader range of providers to ensure that individuals are matched to appropriate services regardless of ability to pay. Specifically, in Canada psychologist and social workers are not universally covered and access often depends on employers benefits, EAPs, or private insurance which commonly cap the number of sessions. By contrast, visits to family doctors and psychiatrist are publicly insured which may explain a shift towards these providers among lower income groups.

Self-perceived mental and general health status were also associated to provider use. Individuals who rated their mental health as poor had significantly higher odds of accessing all four types of mental health providers. However, the findings also reveal potential disparities in access as those who were not visible minorities had higher odds of accessing family doctors and non-Indigenous individuals were more likely to access psychologists. These results align with existing literature documenting racial and cultural inequities in mental health service access in Canada, driven by factors such as discrimination, mistrust of providers, culturally unsafe care, and underrepresentation of racialized and Indigenous practitioners.[30-33] Furthermore, these disparities are also shaped by geography and provider supply. For example, mental health care providers are often concentrated in large urban centre resulting in longer travel times and indirect costs for rural communities to accessing care. [34] These findings underscore the importance of promoting equitable mental health services and support community-based models of care that are grounded in trust and cultural responsiveness. Several actions could improve equitable access to mental health services in Canada. First, expanding publicly funded, evidence-based psychotherapy would reduce the role of financial barriers. Second, integrating mental health care providers into primary care teams by streamlining referrals, provided shared care can result in shortened wait times and improved continuity in care. Equity can be further strengthened through Indigenous-led services, cultural-safety standards, language interpretation, and navigation supports for newcomers and racialized communities. Furthermore, private insurance and EAPs need to be modernized to ensure adequate session coverage. Lastly, further investment needs to be made in community-based mental health care including walk-in counselling, youth hubs, peer and Indigenous led programs and system navigation would expand timely service and reduce avoidable reliance on emergency and specialist services. These findings reveal an inequitable

mental health care system in Canada, where sociodemographic factors such as age, gender, income, education, and racial identity shape not only access but also the type of provider individuals are able to see. While need-based access patterns were evident there are clear structural and systemic barriers that limit equitable engagement with care. These disparities reinforce the importance of expanding publicly funded mental health services beyond primary care and ensuring broader access to psychologists and social workers. As Canada continues to build toward a more integrated and accessible mental health system, efforts must prioritize reducing financial, geographic, and cultural barriers to ensure that all individuals can access the mental health care they need. Future research should explore the intersection of provider preferences, treatment experiences, and long-term outcomes to further inform changes to the mental health care system.

*Future Directions*

To build on these findings, future research should focus on using longitudinal or linked survey administrative data to map sequences of care, switching between providers, and duration/intensity of treatment. Similarly, methods that focus on concurrent mental health service provider use would be valuable allowing for the use of multinomial models and to better understanding how individuals are accessing the mental health care system. Equity should be a focus allowing exploration on intersectional effects across gender, age, income, education, racialization, immigration, Indigenous identity, and urbanicity. Likewise, further exploration is needed on needs and barriers including coverage, out-of-pocket costs, wait times, language, and providers availability. Lastly, qualitative research with equity seeking groups can illuminate preferences, experiences, and structural barriers that quantitative data alone may miss.

*Strengths*

This study has several strengths. First, it leverages the Canadian Community Health Survey across seven cycles (2007–2020), yielding nationally representative estimates and trends, with Statistics Canada's sampling procedures and survey weights enhancing generalizability. Similarly, we use survey-weighted and fit provider-specific models (family doctor, psychiatrist, psychologist, social worker) allowing for a nuanced view of provider specific mental health care. Furthermore, the multivariable models are a priori and equity-focused, adjusting for correlated sociodemographic and health factors to explore variables related to policy.

*Limitations*

This study has several limitations. First, mental health service use was based on self-report and may be subject to recall or reporting biases. Second, only service use within the past year was captured without accounting for frequency, duration, or perceived adequacy of care. Third, the regression models only used cross-sectional data from 2019–2020. This is due to an inability to combine data from different cycles together as predictor variables were measured differently across various cycles of the survey. This means that logistic regression results are not able to describe longer-term patterns. Additionally, service use was measured separately for each provider type, without information on concurrent or integrated care. Likewise, given we did not model concurrent use across providers the results do not capture substitution, sequencing, or intensity of multi-provider care. Additionally, the CCHS lacks questions on visit timing, referral pathways, and number of contacts, limiting our ability to characterize multi-provider pathways. Furthermore, we did not have measures of symptom severity, diagnosis, functional impairment, prior treatment, local provider supply, wait times, out-of-pocket costs, or insurance and EAP coverage. We also lacked information on language, travel time, and referral practices. The lack of these factors does not provide a full picture of mental health service use and the omission of

these factors could confound associations if they correlate with both with both sociodemographic characteristics and provider use. Because of this our findings should be interpreted as adjusted associations rather than causal effects. Future studies with linked longitudinal data and methods that explicitly accommodate overlap is needed.

*Conclusion*

This study provides national evidence on who accesses which mental health providers in Canada and how that varies over time. While primary care remains a central entry point for mental health services, the growing role of psychologists and social workers may reflect changes in public demand and service availability. However, significant disparities persist particularly in access to privately funded services. These findings underscore the need for policy efforts to improve equity in mental health care delivery across provider types and population groups.

**Data Access** The data that support the findings of this study are provided by Statistics Canada microdata files. This study used the Canadian Community Health Survey (CCHS) Public Use Microdata Files (PUMF) for the following cycles: 2007–2008, 2009–2010, 2011–2012, 2013–2014, 2015–2016, 2017–2018, and 2019–2020.

The dataset names are as follows:

1. Canadian Community Health Survey, 2007-2008: Annual Component
2. Canadian Community Health Survey, 2009-2010: Annual Component
3. Canadian Community Health Survey, 2011-2012: Annual Component
4. Canadian Community Health Survey, 2013-2014: Annual Component

5. Canadian Community Health Survey, 2015-2016: Annual Component
6. Canadian Community Health Survey, 2017-2018: Annual Component
7. Canadian Community Health Survey, 2019-2020: Annual Component

Files were accessed through ODESI via the University of Toronto. The R scripts used for data preparation, survey-weighted analyses, and figure generation are available from the corresponding author upon reasonable request for research purposes.

**Acknowledgements:** N/A

**Conflicts of Interest:** All authors report no conflicts of interest.

**Funding:** N/A

**References**

1. Smetanin P, Briante C, Khan M, Stiff D, Ahmad S. The life and economic impact of major mental illnesses in Canada. 2015.

2. Andrews G, Carter, and G L. What people say about their general practitioners' treatment of anxiety and depression. Med J Aust. 2002;176(2):69–69.

3. Lesage A. Prevalence of mental illness and related service untilization in Canada. Canadian Collaborative Mental Health Initiative; 2006.

4. Government of Canada Statistics Canada. Mental health care needs, 2018 [Internet]. 2019 [cited 2025 Mar 30]. Available from: https://www150.statcan.gc.ca/n1/pub/82-625-x/2019001/article/00011-eng.htm

5. Thielke S, Vannoy S, Unützer J. Integrating Mental Health and Primary Care. Prim Care Clin Off Pract. 2007 Sep 1;34(3):571–92.

6. Kates N, Sunderji N, Ng V, Patriquin M, Alloo J, Mirwaldt P, et al. Collaborative Mental Health Care in Canada: Challenges, Opportunities and New Directions. Can J Psychiatry Rev Can Psychiatr. 2023 May;68(5):372–98.

7. Tadic V, Ashcroft R, Brown JB, Dahrouge S. The Role of Social Workers in Interprofessional Primary Healthcare Teams. Healthc Policy. 2020 Aug;16(1):27–42.

8. Bartram M. Income-based inequities in access to mental health services in Canada. Can J Public Health. 2019 Aug 1;110(4):395–403.

9. Satvinder S. Dhingra MPH, Matthew Zack MD, Tara Strine MPH, William S. Pearson PD, Lina Balluz SD. Determining Prevalence and Correlates of Psychiatric Treatment With Andersen's Behavioral Model of Health Services Use. Psychiatr Serv [Internet]. 2010 May 1 [cited 2025 Mar 30]; Available from: https://psychiatryonline.org/doi/10.1176/ps.2010.61.5.524

10. Pei J, Amanvermez Y, Vigo D, Puyat J, Kessler RC, Mortier P, et al. Sociodemographic Correlates of Mental Health Treatment Seeking Among College Students: A Systematic Review and Meta-Analysis. Psychiatr Serv [Internet]. 2024 Jan 31 [cited 2025 Mar 30]; Available from: https://psychiatryonline.org/doi/10.1176/appi.ps.20230414

11. McDonald B, Kulkarni ,Madhura, Andkhoie ,Mustafa, Kendall ,Jeffrey, Gall ,Spencer, Chelladurai ,Shankar, et al. Determinants of self-reported mental health and utilization of mental health services in Canada. Int J Ment Health. 2017 Oct 2;46(4):299–311.

12. Murray JK, Knudson S. Mental health treatment and access for emerging adults in Canada: a systematic review. Front Public Health [Internet]. 2023 Jul 10 [cited 2025 Mar 30];11. Available from: https://www.frontiersin.org/journals/public-health/articles/10.3389/fpubh.2023.1088999/full

13. Steele LS, Dewa CS, Lin E, Lee KLK. Education Level, Income Level and Mental Health Services Use in Canada: Associations and Policy Implications. Healthc Policy. 2007 Aug;3(1):96–106.

14. Slaunwhite AK. The Role of Gender and Income in Predicting Barriers to Mental Health Care in Canada. Community Ment Health J. 2015 Jul 1;51(5):621–7.

15. Alegría M, Zhen-Duan J, O’Malley IS, DiMarzio K. A New Agenda for Optimizing Investments in Community Mental Health and Reducing Disparities. Am J Psychiatry. 2022 Jun;179(6):402–16.

16. Kadel R, Stielke A, Ashton K, Masters R, Dyakova M. Social Return on Investment (SROI) of mental health related interventions—A scoping review. Front Public Health [Internet]. 2022 Dec 9 [cited 2025 Apr 21];10. Available from: https://www.frontiersin.orghttps://www.frontiersin.org/journals/public-health/articles/10.3389/fpubh.2022.965148/full

17. O’Halloran P, O’Connor N. Time to invest in developing community mental health services. Australas Psychiatry. 2016 Jun 1;24(3):268–71.

18. Kates N. Mental Health and Primary Care: Contributing to Mental Health System Transformation in Canada. Can J Commun Ment Health. 2017 Dec;36(4):33–67.

19. MacCarthy D, Weinerman R, Kallstrom L, Kadlec H, Hollander MJ, Patten S. Mental Health Practice and Attitudes of Family Physicians Can Be Changed! Perm J. 2013;17(3):14–7.

20. Mackenzie CS, Erickson J, Deane FP, Wright M. Changes in attitudes toward seeking mental health services: A 40-year cross-temporal meta-analysis. Clin Psychol Rev. 2014 Mar 1;34(2):99–106.

21. Thornicroft G, Sunkel C, Aliev AA, Baker S, Brohan E, Chammay R el, et al. The Lancet Commission on ending stigma and discrimination in mental health. The Lancet. 2022 Oct 22;400(10361):1438–80.

22. Moroz N, Moroz I, D'Angelo MS. Mental health services in Canada: Barriers and cost-effective solutions to increase access. Healthc Manage Forum. 2020 Nov;33(6):282–7.

23. Roberts T, Miguel Esponda G, Krupchanka D, Shidhaye R, Patel V, Rathod S. Factors associated with health service utilisation for common mental disorders: a systematic review. BMC Psychiatry. 2018 Aug 22;18(1):262.

24. Cohen KA, Stiles-Shields C, Winquist N, Lattie EG. Traditional and Nontraditional Mental Healthcare Services: Usage and Preferences Among Adolescents and Younger Adults. J Behav Health Serv Res. 2021 Oct 1;48(4):537–53.

25. Reynolds K. Older adults' pathways to mental health information and treatment: Bridging the gap in knowledge translation. 2016 [cited 2025 Apr 21]; Available from: http://hdl.handle.net/1993/31719

26. Gulliver A, Griffiths KM, Christensen H. Perceived barriers and facilitators to mental health help-seeking in young people: a systematic review. BMC Psychiatry. 2010 Dec 30;10(1):113.

27. Marcus M, Westra H, Mobilizing Minds Research Group. Mental Health Literacy in Canadian Young Adults: Results of a National Survey. Can J Commun Ment Health. 2012 Apr;31(1):1–15.

28. McAllister A, Fritzell S, Almroth M, Harber-Aschan L, Larsson S, Burström B. How do macro-level structural determinants affect inequalities in mental health? – a systematic review of the literature. Int J Equity Health. 2018 Dec 6;17(1):180.

29. Fleury MJ, Ngamini Ngui A, Bamvita JM, Grenier G, Caron J. Predictors of Healthcare Service Utilization for Mental Health Reasons. Int J Environ Res Public Health. 2014 Oct;11(10):10559–86.

30. Faber SC, Osman ,Muna, and Williams MT. Access to mental health care in Canada. Int J Ment Health. 2023 Jul 3;52(3):312–34.

31. Colonna C. Immigrant, Refugee, and Racialized Populations: Social Determinants of Health in Canada (2016 Census). 2019 Feb 22 [cited 2025 Apr 21]; Available from: https://policycommons.net/artifacts/11342405/immigrant-refugee-ethnocultural-and-racialized-populations-and-the-social-determinants-of/12231421/

32. Lai DWL, Li L, Daoust GD. Factors Influencing Suicide Behaviours in Immigrant and Ethno-Cultural Minority Groups: A Systematic Review. J Immigr Minor Health. 2017 Jun;19(3):755–68.

33. Faber SC, Williams MT, Metzger IW, MacIntyre MM, Strauss D, Duniya CG, et al. Lions at the gate: How weaponization of policy prevents people of colour from becoming professional psychologists in Canada. Can Psychol Psychol Can. 2023;64(4):335–5

34. Pang N, Nagata JM, Testa A, Ganson KT. Mapping eating disorders in adolescents and young adults: an investigation of geographic distribution and access to care in Ontario, Canada. Journal of eating disorders. 2024 Sep 9;12(1):136.

Figure 1

Click here to access/download;Figure;Figure 1.tiff

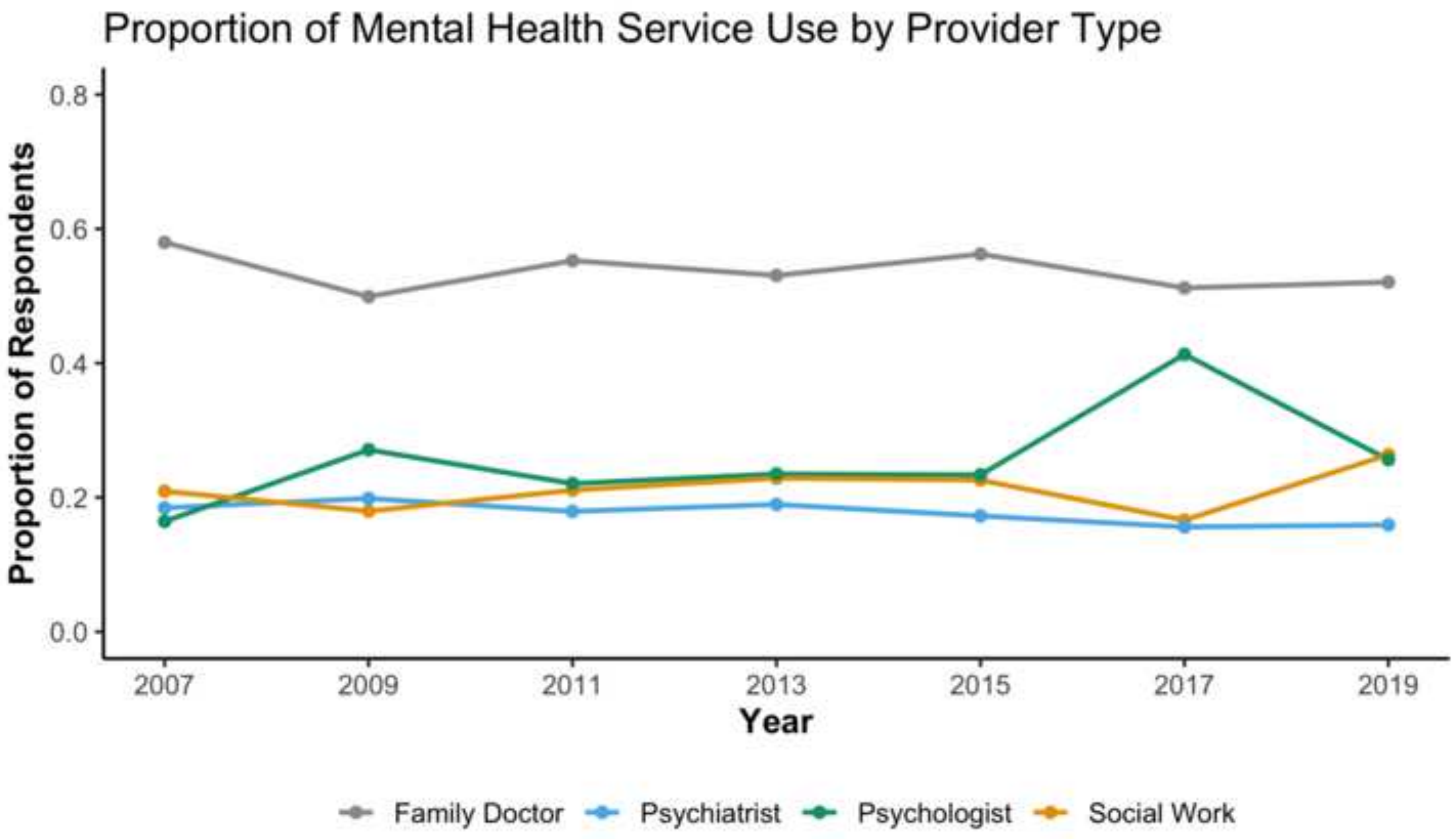

Figure 2

Click here to access/download;Figure;Figure 2.tiff

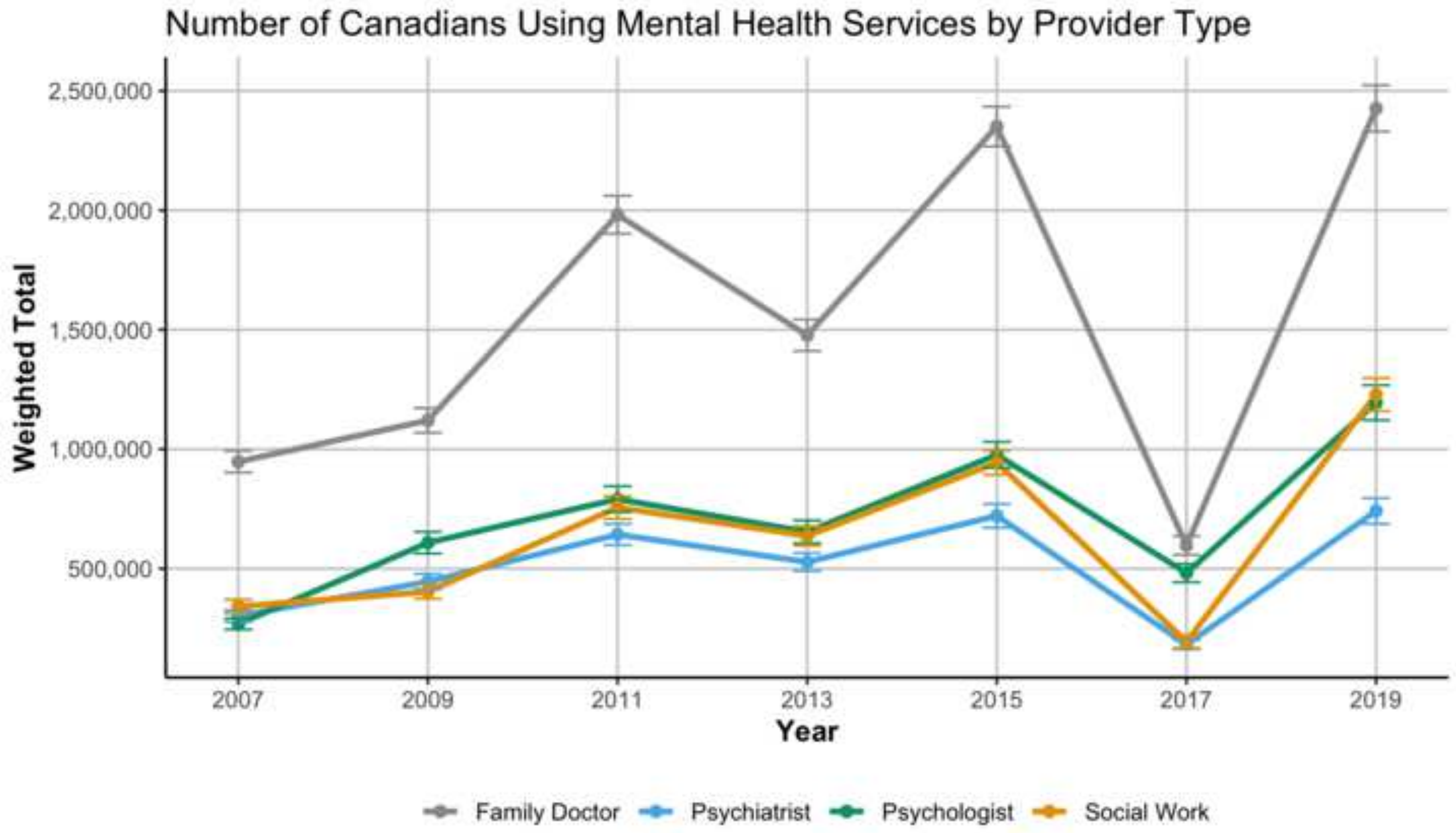

Click here to access/download

**Supporting Information**

Appendix 1 - Variable Definitions and Availability CCHS.docx

Revised Manuscript with Track Changes

# Sociodemographic and Mental Health Predictors of Mental Health Service Use Across Provider Types

Nelson Pang, MSW,[1] Jessie Yeung, MSc[2]

[1] Factor-Inwentash Faculty of Social Work, University of Toronto, Toronto, Ontario, Canada

[2] Department of Statistical Sciences, University of Toronto, Toronto, Ontario, Canada

## Author Note

Correspondence concerning this article should be addressed to Nelson Pang, Factor-Inwentash Faculty of Social Work, University of Toronto, 246 Bloor St W, Toronto, Ontario, Canada, M5S 1V4. Email: nelson.pang@mail.utoronto.ca

**Abstract**

*Objective:* To examine trends in mental health service use across four provider types (family doctors, psychiatrists, psychologists, and social workers) and identify sociodemographic predictors of provider-specific access in Canada.

*Methods:* This study ~~used data from~~analyzes seven cycles (2007–2020) of the Canadian Community Health Survey~~ (CCHS)~~, a nationally representative cross-sectional survey. Trends over time were examined using weighted proportions and counts of service users. Weighted ~~Multivariable~~ multivariable logistic regression models were ~~conducted~~ applied to~~on~~ the 2019–2020 cycle to assess associations between sociodemographic factors and provider-specific service use. ~~Analyses were weighted and adjusted for sex, age, income, education, visible minority status, immigrant status, Aboriginal identity, and self-perceived health.~~

*Results:* Family doctors were consistently the most accessed providers for mental health concerns, followed by psychologists and social workers, with psychiatrists being least accessed. Psychologist and social worker use increased between 2017 and 2019. In the adjusted regression models (2019–2020), women had higher odds of using family doctors (AOR = 1.21, 95% CI: 1.05–1.39) and social workers (1.19, 1.02–1.40) and lower odds of psychiatrists (AOR = 0.66, 95% CI:0.55–0.79) than men. Adults 65+ had greater odds of family-doctor use (AOR = 4.82, 95% CI: 3.59–6.47) and lower odds of psychologist (AOR = 0.33, 95% CI: 0.24–0.45) and social-worker use (AOR = 0.21, 95% CI:0.16–0.29) than ages 12–17. Post-secondary education (vs less than secondary school) was associated with higher psychologist use (AOR = 1.83, 95% CI 1.12–2.98). Higher income (≥$80,000 vs <$20,000) was associated with lower psychiatrist use (AOR = 0.66, 95% CI 0.50–0.86). Non-Indigenous respondents more often used psychologists (AOR = 1.58, 95% CI: 1.13–2.23), and respondents who are not a visible minority

more often used family doctors (AOR = 1.37, 95% CI:1.06–1.77).In regression models, women had significantly higher odds of using family doctors and social workers, while older adults had greater odds of accessing family doctors and lower odds of accessing psychologists and social workers. Higher education was associated with increased psychologist use , whereas lower income and education were linked to family doctor and social work use. Non-Indigenous individuals had higher odds of seeing a psychologist, and those not identifying as a visible minority had higher odds of seeing a family doctor. Poorer perceived mental health was a strong predictor of accessing all provider types.

*Conclusions:* This study reveals a stratified mental health care system in Canada, where sociodemographic factors shape who accesses which providers. While primary care dominates, growth in psychologist and social worker use suggests shifting patterns of engagement. Findings underscore the need for policies that address financial and structural barriers, promote equitable access, and expand coverage for community-based mental health providers.

**Keywords:** Mental Health Care Access, Sociodemographic disparities, Health Care Access, Psychiatric care, Health Service Trends

**Background**

Mental health is a significant public health concern in Canada, with approximately 1 in 5 Canadians experiencing a mental illness every year.[1] Despite this access to mental health care is limited with studies estimating that only around 40–60% of people with a mental health problem

Formatted: Left

~~seeking~~ seek professional assistance.[2,3] In 2018, an estimated 5.3 million Canadians reported needing help for their mental health in the previous year highlighting the widespread demand for mental health support across the Canadian population.[4] Alarmingly, nearly half of these individuals had their needs only partially met (1.2 million) or completely unmet (1.1 million), suggesting substantial gaps in service delivery for mental health. [4]

In Canada, mental health care is provided by a diverse range of professionals, including psychiatrists, psychologists, social workers, nurses, and physicians.[3] These different providers differ not only in their training and scope of practice but also in funding and accessibility.[5] For example, family physicians and psychiatrists are typically publicly funded and can be billed directly to provincial and territorial health insurance plans whereas services provided by psychologists and social workers may require out-of-pocket payment or private insurance coverage.[3] These differences in cost of services may result in financial barriers for individuals seeking care. Furthermore, these differences have implications on who receives care, what kind of care is delivered, and how barriers to access may differ across populations. In recent years, the mental health service landscape in Canada has become increasingly complex with expanding roles in the mental health care for non-physician providers such as psychologists and social workers.[5] This change reflects both efforts to meet growing demand for mental health care, shifts toward evidence-based care, and interprofessional models of service delivery.[6,7]

Previous research has shown that mental health care utilization is shaped by an interplay of need, enabling factors and predisposing factors, such as symptom severity, income, gender, education, cultural background, wait time, and costs.[8-12] Despite Canada having universal health insurance, disparities and inequities are still associated with the underutilization of mental health care services.[8,11,13,14] For example, previous research in Canada has found that individuals with

higher education are more likely to utilize mental health services than those in less educated groups.[11,13] Likewise, previous research has found income-based inequity in access to mental health services.[8,14] Understanding which populations access specific types of providers is critical for informing mental health care and equity-focused policies. For example, investments in community-based providers such as psychologists and social workers may reduce reliance on primary care for mental health, improve continuity of care, and expand culturally appropriate service options.[15-17]

A limitation in this research however is that most studies aggregate all types of mental health providers into one variable limiting ~~for~~ the examination of differences in who accesses ~~what~~ which services and under ~~what~~ which circumstances. As a result, we lack nuanced understanding of how sociodemographic factors shape engagement with specific provider types, such as psychologists or social workers, whose roles and funding structures differ significantly from psychiatrists and family doctors. Furthermore, few studies have examined national level trends over time in provider specific mental health service use limiting our ability to assess how patterns have shifted in response to evolving service landscapes, policy changes, or public attitudes towards mental health. To address these gaps, ~~Additionally, because of this aggregation there are limited studies that examine trends overtime on how Canadians engage with different mental health care providers. T~~this study focuses on examining how sociodemographic and mental health characteristics are associated with mental health service use across four provider types (family doctors, psychiatrists, psychologists, and social workers) and trends in their use over time in Canada. By disaggregating service use by provider type, this research aims to uncover distinct access patterns across sociodemographic groups and inform efforts to promote

equity in mental health care.~~This research will addresses key knowledge gaps by disaggregating provider-specific service use and examine how these patterns vary across populations and years.~~

**Methods**

*Study Design and Data Source*

We conducted a cross-sectional, population-based analysis using seven cycles (2007-2020) of the Canadian Community Health Survey (CCHS) public-use microdata. The CCHS is a nationally representative cross-sectional survey administered by Statistics Canada. The CCHS is a self-reported survey on health, healthcare utilization, and health determinants of the Canadian population aged 12 and above. The CCHS covers the population 12 years of age and over who live in Canada. Excluded from the CCHS are institutionalized residents, full-time members of the Canadian Armed Forces, residents of certain remote regions, or people living on reserves/other Indigenous settlements in the provinces. Altogether, these exclusions represent less than 3% of the target population. Sample sizes varied across cycles, and all analyses applied survey weights provided by Statistics Canada to ensure representativeness of the Canadian population. This study summarized trends of provider specific mental health access across 2007–2020 and provider specific logistic regressions were restricted to 2019–2020 because it was the most recent cycle and contained key predictors specified a priori for multivariable regression. See Appendix 1 for details of variable availability and wording between CCHS cycles.

~~This study draws from seven cycles of the Canadian Community Health Survey (CCHS) collected between 2007 and 2020. The CCHS is a nationally representative cross-sectional survey administered by Statistics Canada. The CCHS is a self-reported survey on health, healthcare utilization, and health determinants of the Canadian population aged 12 and above.~~

~~Sample sizes varied across cycles, and all analyses applied survey weights provided by Statistics Canada to ensure representativeness of the Canadian population.~~

*Ethics, Data Access, and Participant Confidentiality*

This study used retrospective survey data from the CCHS Public Use Microdata Files (PUMFs), accessed for research purposes in January 2025. These data are collected and publicly released by Statistics Canada, which obtains informed consent from participants at the time of original data collection. The PUMFs are fully anonymized prior to release and do not contain any identifying information. As a result, the authors did not have access to any information that could identify individual participants during or after data collection, and additional institutional ethics approval was not required for this secondary analysis.

*Outcome variables*

The data was subsetted to only include respondents who have consulted a mental health professional in the past 12-months (n=13,580/weighted n= 4,679,132.44). The primary outcome were binary indicators of if respondents reported having seen or talked to a specific health professional based on the question *"In the past 12 months have you seen or talked to a health professional about your emotional or mental health?"*. The analysis focused on the four most common providers for mental health care (family doctor, psychiatrist, psychologist, and social work). Each provider was coded as a separate variable as respondents could see more than one provider.

*Predictor variables*

A variety of sociodemographic and mental health characteristics were included as predictors in the model. Sociodemographic characteristics included sex, age, household income,

being a visible minority ~~status~~, education, immigration status, and Aboriginal identity. Mental health characteristics included perceived health and perceived mental health.

*Statistical Analysis*

An exploratory descriptive analysis was conducted to examine trends in mental health service use across provider types over time (2007-2020). To identify factors associated with provider-specific mental health service we fit four separate, survey-weighted multivariable logistic regression models ~~use four separate logistic regression models were conducted~~ using the 2019-2020 cycle of the data for each provider type (family doctor, psychiatrist, psychologist, and social worker). ~~Each model assessed~~The models access the relationship between ~~the outcome variables and~~ the ~~likelihood~~ use of ~~using that~~a specific provider adjusting for all other covariates. Each model included the same a priori covariates listed above to keep models comparable. Outcomes were binary (any past-12-month contact with the provider: yes/no). We modeled providers separately because respondents may see multiple providers and access differs for various reasons including pathways and financing. A single multinomial model would also impose inappropriate mutual exclusivity. Two-tailed tests with a threshold of $\alpha = 0.05$ were used for all statistical tests. All analyses were conducted using R statistical software and survey weights were applied to account for the complex sampling design of the CCHS and to be representative of the Canadian population.

**Results**

Between 2007 and 2020, family doctors consistently accounted for the highest proportion of mental health service use in Canada in every cycle with over half of service users responding to having accessed a family doctor for mental health care (See Figure 1). Across all cycles, the rank order of providers was stable and the pattern over the years are relatively stable, with only

modest year-to-year variation The proportion of people who accessed care through psychologists and social workers showed a gradual increase over time, while the proportion of visits to psychiatrists remained relatively unchanged over time. A one-year divergence appears in 2017, with a temporary spike for psychologist and dips slightly for the other providers but by the next cycle (2019) the pattern returns with psychologist and social workers converging at similar levels. We interpret 2017 as single cycle fluctuation rather than a break in trend. Estimates are survey-weighted and reflect that provider contacts are not mutually exclusive.

~~In 2017, there was a significant increase in the proportion of those who accessed a psychologist potentially reflecting data issues in the 2017 cycle of the data.~~

Formatted: Indent: First line: 0"

*Figure 1. Past-12-month contact for mental health by provider, CCHS 2007–2019 (survey-weighted proportion)~~Trends in Proportion of Mental Health Service Use by Provider Type~~*

When examining weighted estimates of the number of Canadians accessing care, similar trends were observed (See Figure 2). The total number of individuals seeing family doctors for mental health reasons far exceeded those accessing psychologists, psychiatrists, or social workers. However, all provider types showed increases in absolute numbers over time reflecting increased mental service use overtime. Notably, a temporary drop in service use was observed in 2017 with a rebound by 2019 across all provider types, potentially reflecting data collection limitations in that survey cycle. Given each cycle of the CCHS has different weight calibrations and sample allocations, we believe this is a cycle level fluctuation rather than a structural change to mental health care use.

*Figure 2. Weighted number of Canadians reporting past-12-month mental-health contact by provider, CCHS 2007–2019*~~*Weighted Number of Canadians Using Mental Health Services by Provider Type*~~

Adjusted ~~Associations~~ associations between sociodemographic and mental health characteristics and provider-specific mental health service use are presented in Table 1. Because outcomes were modeled separately the estimates below should be interpreted as provider-specific associations. ~~Women had significantly higher odds than men of using social work (OR = 1.19, 95% CI: 1.02–1.40, p = 0.028) and family doctor services (OR = 1.21, 95% CI: 1.05–1.39, p = 0.007), but lower odds of seeing a psychiatrist (OR = 0.66, 95% CI: 0.55–0.79, p < .001).~~

Table 1. Adjusted Associations between Sociodemographic Variables and Mental Health ~~Service~~ Provider Specific Mental Health Service Use

| | Social Work | | | Family Doctor | | | Psychiatry | | | Psychologist | | |
|---|---|---|---|---|---|---|---|---|---|---|---|---|
| | **AOR** | **95% CI** | **p-value** | **AOR** | **95% CI** | **p-value** | **AOR** | **95% CI** | **p-value** | **AOR** | **95% CI** | **p-value** |
| **Sex** (Ref: Men) | | | | | | | | | | | | |
| Women | **1.19** | **1.02, 1.4** | **0.028** | **1.21** | **1.05, 1.39** | **0.007** | **0.66** | **0.55, 0.79** | **<.001** | 0.98 | 0.84, 1.14 | 0.760 |
| **Age** (Ref: 12-17) | | | | | | | | | | | | |
| 18-34 | **0.49** | **0.38, 0.63** | **<.001** | **2.97** | **2.26, 3.91** | **<.001** | 0.82 | 0.55, 1.22 | 0.325 | 0.78 | 0.59, 1.03 | 0.084 |
| 35-49 | **0.38** | **0.3, 0.49** | **<.001** | **3.74** | **2.86, 4.9** | **<.001** | 0.62 | 0.42, 0.91 | 0.014 | 0.93 | 0.71, 1.22 | 0.606 |
| 50-64 | **0.25** | **0.18, 0.33** | **<.001** | **3.98** | **3.01, 5.26** | **<.001** | 0.83 | 0.56, 1.22 | **0.343** | **0.69** | **0.52, 0.92** | **0.011** |

| | | | | | | | | | | | | |
|---|---|---|---|---|---|---|---|---|---|---|---|---|
| 65+ | **0.21** | **0.16, 0.29** | **<.001** | **4.82** | **3.59, 6.47** | **<.001** | 0.96 | 0.64, 1.44 | **0.839** | **0.33** | **0.24, 0.45** | **<.001** |
| **Household Income**<br>(Ref: No income or less than $20,000) | | | | | | | | | | | | |
| $20,000 to $39,999 | 0.95 | 0.69, 1.31 | 0.759 | **1.37** | **1.04, 1.81** | **0.027** | 0.82 | 0.6, 1.12 | 0.203 | 0.87 | 0.62, 1.23 | 0.444 |
| $40,000 to $59,999 | 0.89 | 0.65, 1.22 | 0.478 | **1.53** | **1.15, 2.03** | **0.004** | **0.7** | **0.5, 0.97** | **0.031** | 0.93 | 0.67, 1.31 | 0.694 |
| $60,000 to $79,999 | 0.82 | 0.59, 1.14 | 0.234 | 1.32 | 0.98, 1.77 | 0.070 | **0.6** | **0.42, 0.85** | **0.004** | 1.14 | 0.77, 1.68 | 0.507 |
| $80,000 or more | 0.90 | 0.68, 1.19 | 0.472 | **1.41** | **1.11, 1.8** | **0.006** | **0.66** | **0.5, 0.86** | **0.002** | 1.09 | 0.82, 1.46 | 0.546 |
| **Visible Minority**<br>(Ref: Visible Minority) | | | | | | | | | | | | |
| Not a visible minority | 1.05 | 0.79, 1.41 | 0.717 | **1.37** | **1.06, 1.77** | **0.017** | 1.23 | 0.85, 1.8 | 0.270 | 1.2 | 0.88, 1.63 | 0.245 |
| **Highest level of education in household**<br>(Ref: Less than secondary school graduation) | | | | | | | | | | | | |
| Secondary school graduation, no post-secondary education | 0.96 | 0.62, 1.49 | 0.862 | 0.76 | 0.54, 1.08 | 0.128 | 1.12 | 0.73, 1.72 | 0.598 | 1.28 | 0.74, 2.24 | 0.378 |
| Post-secondary certificate/diploma / university degree | 1.08 | 0.72, 1.62 | 0.706 | **0.61** | **0.45, 0.83** | **0.002** | 1.4 | 0.95, 2.08 | 0.093 | **1.83** | **1.12, 2.98** | **0.016** |
| **Aboriginal identity - First nations / Metis / Inuk (Inuit)**<br>(Ref: Yes) | | | | | | | | | | | | |
| No | 0.84 | 0.63, 1.13 | 0.262 | 1.12 | 0.84, 1.49 | 0.423 | 1.1 | 0.75, 1.61 | 0.636 | **1.58** | **1.13, 2.23** | **0.008** |
| **Immigrant**<br>(Ref: Landed immigrant / non-permanent resident) | | | | | | | | | | | | |
| Non-immigrant (Canadian born) | 1 | 0.77, 1.29 | 0.990 | 1 | 0.8, 1.26 | 0.970 | 1.27 | 0.95, 1.7 | 0.111 | 0.93 | 0.71, 1.21 | 0.567 |
| **Perceived Health**<br>(Ref: Poor) | | | | | | | | | | | | |

| | | | | | | | | | | | | |
|---|---|---|---|---|---|---|---|---|---|---|---|---|
| Fair | 0.74 | 0.53, 1.04 | 0.086 | 0.9 | 0.66, 1.23 | 0.509 | **0.68** | **0.48, 0.94** | **0.022** | 1.22 | 0.85, 1.76 | 0.284 |
| Good | 0.73 | 0.53, 1 | 0.051 | 1.02 | 0.76, 1.37 | 0.883 | **0.56** | **0.41, 0.77** | **<.001** | 1.24 | 0.89, 1.72 | 0.200 |
| Very good | 0.73 | 0.52, 1.02 | 0.062 | 0.91 | 0.67, 1.24 | 0.553 | **0.39** | **0.27, 0.55** | **<.001** | 1.33 | 0.94, 1.88 | 0.102 |
| Excellent | 0.72 | 0.49, 1.07 | 0.103 | 0.78 | 0.56, 1.11 | 0.165 | **0.34** | **0.22, 0.52** | **<.001** | **1.47** | **1.01, 2.15** | **0.045** |
| **Perceived Mental Health** (Ref: Poor) | | | | | | | | | | | | |
| Fair | 0.79 | 0.59, 1.05 | 0.101 | 0.91 | 0.68, 1.23 | 0.553 | **0.52** | **0.38, 0.7** | **<.001** | 0.93 | 0.66, 1.3 | 0.662 |
| Good | **0.65** | **0.49, 0.87** | **0.004** | 0.74 | **0.55, 1** | **0.050** | **0.33** | **0.24, 0.44** | **<.001** | 0.92 | 0.66, 1.28 | 0.618 |
| Very good | **0.63** | **0.46, 0.86** | **0.004** | 0.57 | **0.42, 0.78** | **<.001** | **0.28** | **0.2, 0.41** | **<.001** | 0.9 | 0.63, 1.28 | 0.559 |
| Excellent | **0.64** | **0.44, 0.94** | **0.024** | 0.58 | **0.41, 0.84** | **0.003** | **0.24** | **0.16, 0.37** | **<.001** | 0.84 | 0.56, 1.26 | 0.400 |
| (Intercept) | 1.77 | 0.9, 3.5 | 0.101 | **0.33** | **0.18, 0.61** | **<.001** | 1.02 | 0.47, 2.24 | 0.951 | **0.13** | **0.06, 0.28** | **<.001** |

Note. AOR: Adjusted Odds Ratio; CI: Confidence ~~Ratio~~Interval; **Boldface** indicates statistical significance at the 0.05 level.

**Formatted:** Font: Bold

**Formatted:** Font: Bold

~~Older age was associated with increased use of family doctors. Specifically, those age 65+ had the highest odds (OR = 4.82, 95% CI: 3.59–6.47, p < .001), but lowest odds of using social workers (OR = 0.21, 95% CI: 0.16–0.29, p < .001) and psychologists (OR = 0.33, 95% CI: 0.24–0.45, p < .001). For household income, individuals in all income groups over $40,000 had significantly lower odds of accessing psychiatrist. Notably, those in the highest income group ($80,000+) had lower odds of seeing a psychiatrist (OR = 0.66, 95% CI: 0.50–0.86, p = 0.002) compared to the lowest income group category. In contrast, individuals in higher income groups has higher odds of accessing a family doctor for mental health. Conspicuously, there were no~~

significant associations found between income and psychologist use. Additionally, educational attainment was significantly associated with psychologist use. Compared to households with no high school attainment, respondents in households with a post-secondary degree had higher odds of accessing psychologists (OR = 1.83, 95% CI: 1.12–2.98, p = 0.016), but lower odds of accessing family doctors (OR = 0.61, 95% CI: 0.45–0.83, p = 0.002).

Few significant associations emerged for immigrant status or Indigenous identity, with the exception that non-Indigenous respondents had significantly higher odds of psychologist use (OR = 1.58, 95% CI: 1.13–2.23, p = 0.008). Similarly, not being a visible minority was associated with higher odds of seeing a family doctor (OR 1.37, 95% CI: 1.06-1.77, p=0.017) but no other provider use was significant.

Perceived mental health was a consistent and strong predictor of provider use. Individuals reporting "excellent" perceived mental health had significantly lower odds of seeing a psychiatrist (OR = 0.24, 95% CI: 0.16–0.37, p < .001), family doctor (OR = 0.58, 95% CI: 0.41–0.84, p = 0.003), or social worker (OR = 0.64, 95% CI: 0.44–0.94, p = 0.024), compared to those reporting poor mental health.

Use of social workers was higher among women (AOR = 1.19, 95% CI: 1.02–1.40) and decreased with age across all groups relative to the 12–17 group (AOR [18–34] = 0.49, 95% CI: 0.38–0.63; AOR[35–49] = 0.38, 95% CI: 0.30–0.49; AOR [50–64] = 0.25, 95% CI: 0.18–0.33; AOR [65+] = 0.21, 95% CI: 0.16–0.29). Better perceived mental health was associated with lower odds compared to having poor perceived mental health (AOR [good] = 0.65, 95% CI: 0.49–0.87; AOR [very good] = 0.63, 95% CI: 0.46–0.86; AOR [excellent] = 0.64, 95% CI: 0.44–0.94).

Use of family doctors was higher among women than men (AOR = 1.21, 95% CI: 1.05–1.39) and increased sharply with age (vs. 12–17: AOR [18–34] = 2.97, 95% CI: 2.26–3.91; AOR[35–49] = 3.74, 95% CI: 2.86–4.90; AOR [50–64] = 3.98, 95% CI: 3.01–5.26; AOR [65+] = 4.82, 95% CI: 3.59–6.47). Higher household income was associated with higher odds of using a family doctor (vs. No income or less than $20,000. AOR[$40–59k]: 1.53, 95% CI:1.15–2.03; AOR[ ≥$80k]= 1.41, 95% CI: 1.11–1.80), whereas post-secondary education was associated with lower odds compared to those with less than secondary school graduation (AOR = 0.61, 95% CI: 0.45–0.83). Not being a visible minority was also associated with higher odds of seeing a family doctor (AOR: 1.37, 95% CI: 1.06–1.77). Better perceived mental health related to lower use compared to having poor perceived mental health (AOR [very good] = 0.57, 95% CI: 0.42–0.78; AOR [excellent] = 0.58, 95% CI: 0.41–0.84).

Odds of seeing a psychiatrist were lower for women than men (AOR = 0.66, 95% CI: 0.55–0.79) and lower for adults aged 35–49 relative to 12–17 (AOR = 0.62, 95% CI: 0.42–0.91). Higher income was associated with lower odds (vs. No income or less than $20,000. AOR [$40–59k] = 0.70, 95% CI: 0.50–0.97; AOR [$60–79k] = 0.60, 95% CI:0.42–0.85; APR [≥$80k] = 0.66, 95% CI: 0.50–0.86). Better perceived health and perceived mental health related to lower use (e.g., perceived health excellent vs poor perceived health. AOR = 0.34, 95% CI; 0.22–0.52; perceived mental health excellent. AOR = 0.24, 95% CI: 0.16–0.37).

Psychologist use declined at older ages (AOR [50–64] = 0.69, 95% CI:0.52–0.92; AOR [65+] = 0.33, 95% CI: 0.24–0.45) compared with the 12–17 age group. In contrast, post-secondary education was associated with higher odds (AOR =1.83, 95% CI: 1.12–2.98) compared to those with less than secondary school graduation. Being non-indigenous was

associated with higher psychologist used compared to indigenous groups (AOR: 1.58, 95% CI: 1.13–2.23). Excellent perceived health was also associated with higher odds compared to those with poor perceived health (AOR: 1.47, 95% CI: 1.01–2.15).

Across providers, immigrant status was not significantly associated with mental health service use. Household income showed no association with psychologist or social worker use. Being a visible minority and Indigenous identity showed no associations overall, with two exceptions (respondents who were not a visible minority having higher odds of family-doctor use and non-Indigenous respondents having higher odds of psychologist use).

**Formatted:** Indent: First line: 0.5"

## Discussion

This study examined national trends in mental health service use across four provider types (family doctors, psychiatrists, psychologists, and social workers) and identified sociodemographic predictors to accessing each provider using a recent nationally representative survey. Results show that family doctors were the most common provider for mental health, followed by psychologists and social workers, while psychiatrists are accessed less frequently. These trends were generally stable over time, with increases in psychologist and social worker use observed between 2017 and 2019. Consistent with previous research, family doctors were found to be the most commonly accessed mental health care provider, reaffirming the central role of primary care in Canada's mental health system.[5,18] Given the high rates and numbers of people who seek mental health care from family doctors it is important that family doctors are trained in assessment and treatment of mental health.[19] Access to psychologists and social workers increased in recent years, particularly between 2017 and 2019, which may reflect

changing public attitudes toward mental health, reduced stigma, and increased availability of services.[20-22]

Logistic regression analyses from the 2019–2020 CCHS cycle indicate significant differences in access to providers across different demographic groups. Women had higher odds of seeking support from family doctors and social workers compared to men, aligning with prior literature showing greater mental health service use among women.[23] Age emerged as a significant predictor of provider type with older adults more likely to seek care from family doctors and younger individuals more likely to access psychologists and social workers. This trend may reflect generational differences in mental health literacy, familiarity with provider types, and access pathways.[24,25] Additionally, mental health services delivered by psychologists and social workers are not universally covered by provincial health plans in Canada, and younger individuals may access these providers through post-secondary institutions, school-based programs, or employee insurance plans. The rise in mental health awareness among younger generations, combined with greater comfort in discussing psychological issues, may also increase their likelihood of seeking specialized care.[26,27] In contrast, older adults may be more affected by stigma surrounding mental illness and thus prefer family doctors who can address mental and physical health simultaneously in a less stigmatizing setting.[25] Furthermore, these age patterns likely also reflect differences in care continuity and navigation. Older adults have stronger ties to a usual family doctor and more multimorbidity which makes primary care a natural entry point for mental and physical concerns. Younger adults in contrast more often have access to private insurance and Employee Assistance Programs (EAP) for counselling which may lower the threshold to see psychologist and social workers.

**Formatted:** Font: Not Bold

Income and education were associated with distinct patterns of provider use. Individuals with higher education were more likely to access psychologists, potentially reflecting the private-pay nature of these services and barriers related to insurance coverage. In contrast, individuals with lower income and education levels were more likely to see family doctors or social workers, who are often covered under public or community-based services. These patterns highlight ongoing structural inequities in access to mental health care, where the ability to pay often determines the type and intensity of support received.[28,29] These findings support the need for publicly funded access to a broader range of providers to ensure that individuals are matched to appropriate services regardless of ability to pay. Specifically, in Canada psychologist and social workers are not universally covered and access often depends on employers benefits, EAPs, or private insurance which commonly cap the number of sessions. By contrast, visits to family doctors and psychiatrist are publicly insured which may explain a shift towards these providers among lower income groups.

Self-perceived mental and general health status were also associated to provider use. Individuals who rated their mental health as poor had significantly higher odds of accessing all four types of mental health providers. However, the findings also reveal potential disparities in access as those who were not visible minorities had higher odds of accessing family doctors and non-Indigenous individuals were more likely to access psychologists. These results align with existing literature documenting racial and cultural inequities in mental health service access in Canada, driven by factors such as discrimination, mistrust of providers, culturally unsafe care, and underrepresentation of racialized and Indigenous practitioners.[30-33] Furthermore, these disparities are also shaped by geography and provider supply. For example, mental health care providers are often concentrated in large urban centre resulting in longer travel times and indirect

costs for rural communities to accessing care.[34] These findings underscore the importance of promoting equitable mental health services and support community-based models of care that are grounded in trust and cultural responsiveness. Several actions could improve equitable access to mental health services in Canada. First, expanding publicly funded, evidence-based psychotherapy would reduce the role of financial barriers. Second, integrating mental health care providers into primary care teams by streamlining referrals, provided shared care can result in shortened wait times and improved continuity in care. Equity can be further strengthened through Indigenous-led services, cultural-safety standards, language interpretation, and navigation supports for newcomers and racialized communities. Furthermore, private insurance and EAPs need to be modernized to ensure adequate session coverage. Lastly, further investment needs to be made in community-based mental health care including walk-in counselling, youth hubs, peer and Indigenous led programs and system navigation would expand timely service and reduce avoidable reliance on emergency and specialist services.

These findings reveal an inequitable mental health care system in Canada, where sociodemographic factors such as age, gender, income, education, and racial identity shape not only access but also the type of provider individuals are able to see. While need-based access patterns were evident there are clear structural and systemic barriers that limit equitable engagement with care. These disparities reinforce the importance of expanding publicly funded mental health services beyond primary care and ensuring broader access to psychologists and social workers. As Canada continues to build toward a more integrated and accessible mental health system, efforts must prioritize reducing financial, geographic, and cultural barriers to ensure that all individuals can access the mental health care they need. Future research should

**Formatted:** Indent: First line: 0.5"

explore the intersection of provider preferences, treatment experiences, and long-term outcomes to further inform changes to the mental health care system.

*Future Directions*

To build on these findings, future research should focus on using longitudinal or linked survey administrative data to map sequences of care, switching between providers, and duration/intensity of treatment. Similarly, methods that focus on concurrent mental health service provider use would be valuable allowing for the use of multinomial models and to better understanding how individuals are accessing the mental health care system. Equity should be a focus allowing exploration on intersectional effects across gender, age, income, education, racialization, immigration, Indigenous identity, and urbanicity. Likewise, further exploration is needed on needs and barriers including coverage, out-of-pocket costs, wait times, language, and providers availability. Lastly, qualitative research with equity seeking groups can illuminate preferences, experiences, and structural barriers that quantitative data alone may miss.

*Strengths*

This study has several strengths. First, it leverages the Canadian Community Health Survey across seven cycles (2007–2020), yielding nationally representative estimates and trends, with Statistics Canada's sampling procedures and survey weights enhancing generalizability. Similarly, we use survey-weighted and fit provider-specific models (family doctor, psychiatrist, psychologist, social worker) allowing for a nuanced view of provider specific mental health care. Furthermore, the multivariable models are a priori and equity-focused, adjusting for correlated sociodemographic and health factors to explore variables related to policy.

*Limitations*

**Formatted:** Font: Italic

**Formatted:** Indent: First line: 0.5"

**Formatted:** Font: Not Italic

This study has several limitations. First, mental health service use was based on self-report and may be subject to recall or reporting biases. Second, only service use within the past year was captured without accounting for frequency, duration, or perceived adequacy of care. Third, the regression models only used cross-sectional data from 2019–2020. This is due to an inability to combine data from different cycles together as predictor variables were measured differently across various cycles of the survey. This means that logistic regression results are not able to describe longer-term patterns. Additionally, service use was measured separately for each provider type, without information on concurrent or integrated care. Likewise, given we did not model concurrent use across providers the results do not capture substitution, sequencing, or intensity of multi-provider care. Additionally, the CCHS lacks questions on visit timing, referral pathways, and number of contacts, limiting our ability to characterize multi-provider pathways. Furthermore, we did not have measures of symptom severity, diagnosis, functional impairment, prior treatment, local provider supply, wait times, out-of-pocket costs, or insurance and EAP coverage. We also lacked information on language, travel time, and referral practices. The lack of these factors does not provide a full picture of mental health service use and the omission of these factors could confound associations if they correlate with both with both sociodemographic characteristics and provider use. Because of this our findings should be interpreted as adjusted associations rather than causal effects. Future studies with linked longitudinal data and methods that explicitly accommodate overlap is needed.

*Conclusion*

This study provides national evidence on who accesses which mental health providers in Canada and how that varies over time. While primary care remains a central entry point for mental health services, the growing role of psychologists and social workers may reflect changes

in public demand and service availability. However, significant disparities persist particularly in access to privately funded services. These findings underscore the need for policy efforts to improve equity in mental health care delivery across provider types and population groups.

**Data Access** The data that support the findings of this study are provided by Statistics Canada microdata files. This study used the Canadian Community Health Survey (CCHS) Public Use Microdata Files (PUMF) for the following cycles: 2007–2008, 2009–2010, 2011–2012, 2013–2014, 2015–2016, 2017–2018, and 2019–2020.

**Formatted:** Font: Bold

The dataset names are as follows:

1. Canadian Community Health Survey, 2007-2008: Annual Component
2. Canadian Community Health Survey, 2009-2010: Annual Component
3. Canadian Community Health Survey, 2011-2012: Annual Component
4. Canadian Community Health Survey, 2013-2014: Annual Component
5. Canadian Community Health Survey, 2015-2016: Annual Component
6. Canadian Community Health Survey, 2017-2018: Annual Component
7. Canadian Community Health Survey, 2019-2020: Annual Component

Files were accessed through ODESI via the University of Toronto. The R scripts used for data preparation, survey-weighted analyses, and figure generation are available from the corresponding author upon reasonable request for research purposes.

~~The data that support the findings of this study are provided by Statistics Canada microdata files.~~

**Acknowledgements:** N/A

**Conflicts of Interest:** All authors report no conflicts of interest.

**Funding:** N/A

## References

1. Smetanin P, Briante C, Khan M, Stiff D, Ahmad S. The life and economic impact of major mental illnesses in Canada. 2015.
2. Andrews G, Carter, and G L. What people say about their general practitioners' treatment of anxiety and depression. Med J Aust. 2002;176(2):69–69.
3. Lesage A. Prevalence of mental illness and related service untilization in Canada. Canadian Collaborative Mental Health Initiative; 2006.
4. Government of Canada Statistics Canada. Mental health care needs, 2018 [Internet]. 2019 [cited 2025 Mar 30]. Available from: https://www150.statcan.gc.ca/n1/pub/82-625-x/2019001/article/00011-eng.htm
5. Thielke S, Vannoy S, Unützer J. Integrating Mental Health and Primary Care. Prim Care Clin Off Pract. 2007 Sep 1;34(3):571–92.
6. Kates N, Sunderji N, Ng V, Patriquin M, Alloo J, Mirwaldt P, et al. Collaborative Mental Health Care in Canada: Challenges, Opportunities and New Directions. Can J Psychiatry Rev Can Psychiatr. 2023 May;68(5):372–98.
7. Tadic V, Ashcroft R, Brown JB, Dahrouge S. The Role of Social Workers in Interprofessional Primary Healthcare Teams. Healthc Policy. 2020 Aug;16(1):27–42.

8. Bartram M. Income-based inequities in access to mental health services in Canada. Can J Public Health. 2019 Aug 1;110(4):395–403.

9. Satvinder S.  Dhingra MPH, Matthew Zack MD, Tara Strine MPH, William S.  Pearson PD, Lina Balluz SD. Determining Prevalence and Correlates of Psychiatric Treatment With Andersen's Behavioral Model of Health Services Use. Psychiatr Serv [Internet]. 2010 May 1 [cited 2025 Mar 30]; Available from: https://psychiatryonline.org/doi/10.1176/ps.2010.61.5.524

10. Pei J, Amanvermez Y, Vigo D, Puyat J, Kessler RC, Mortier P, et al. Sociodemographic Correlates of Mental Health Treatment Seeking Among College Students: A Systematic Review and Meta-Analysis. Psychiatr Serv [Internet]. 2024 Jan 31 [cited 2025 Mar 30]; Available from: https://psychiatryonline.org/doi/10.1176/appi.ps.20230414

11. McDonald B, Kulkarni ,Madhura, Andkhoie ,Mustafa, Kendall ,Jeffrey, Gall ,Spencer, Chelladurai ,Shankar, et al. Determinants of self-reported mental health and utilization of mental health services in Canada. Int J Ment Health. 2017 Oct 2;46(4):299–311.

12. Murray JK, Knudson S. Mental health treatment and access for emerging adults in Canada: a systematic review. Front Public Health [Internet]. 2023 Jul 10 [cited 2025 Mar 30];11. Available from: https://www.frontiersin.org/journals/public-health/articles/10.3389/fpubh.2023.1088999/full

13. Steele LS, Dewa CS, Lin E, Lee KLK. Education Level, Income Level and Mental Health Services Use in Canada: Associations and Policy Implications. Healthc Policy. 2007 Aug;3(1):96–106.

14. Slaunwhite AK. The Role of Gender and Income in Predicting Barriers to Mental Health Care in Canada. Community Ment Health J. 2015 Jul 1;51(5):621–7.

15. Alegría M, Zhen-Duan J, O'Malley IS, DiMarzio K. A New Agenda for Optimizing Investments in Community Mental Health and Reducing Disparities. Am J Psychiatry. 2022 Jun;179(6):402–16.

16. Kadel R, Stielke A, Ashton K, Masters R, Dyakova M. Social Return on Investment (SROI) of mental health related interventions—A scoping review. Front Public Health [Internet]. 2022 Dec 9 [cited 2025 Apr 21];10. Available from: https://www.frontiersin.orghttps://www.frontiersin.org/journals/public-health/articles/10.3389/fpubh.2022.965148/full

17. O'Halloran P, O'Connor N. Time to invest in developing community mental health services. Australas Psychiatry. 2016 Jun 1;24(3):268–71.

18. Kates N. Mental Health and Primary Care: Contributing to Mental Health System Transformation in Canada. Can J Commun Ment Health. 2017 Dec;36(4):33–67.

19. MacCarthy D, Weinerman R, Kallstrom L, Kadlec H, Hollander MJ, Patten S. Mental Health Practice and Attitudes of Family Physicians Can Be Changed! Perm J. 2013;17(3):14–7.

20. Mackenzie CS, Erickson J, Deane FP, Wright M. Changes in attitudes toward seeking mental health services: A 40-year cross-temporal meta-analysis. Clin Psychol Rev. 2014 Mar 1;34(2):99–106.

21. Thornicroft G, Sunkel C, Aliev AA, Baker S, Brohan E, Chammay R el, et al. The Lancet Commission on ending stigma and discrimination in mental health. The Lancet. 2022 Oct 22;400(10361):1438–80.

22. Moroz N, Moroz I, D'Angelo MS. Mental health services in Canada: Barriers and cost-effective solutions to increase access. Healthc Manage Forum. 2020 Nov;33(6):282–7.

23. Roberts T, Miguel Esponda G, Krupchanka D, Shidhaye R, Patel V, Rathod S. Factors associated with health service utilisation for common mental disorders: a systematic review. BMC Psychiatry. 2018 Aug 22;18(1):262.

24. Cohen KA, Stiles-Shields C, Winquist N, Lattie EG. Traditional and Nontraditional Mental Healthcare Services: Usage and Preferences Among Adolescents and Younger Adults. J Behav Health Serv Res. 2021 Oct 1;48(4):537–53.

25. Reynolds K. Older adults' pathways to mental health information and treatment: Bridging the gap in knowledge translation. 2016 [cited 2025 Apr 21]; Available from: http://hdl.handle.net/1993/31719

26. Gulliver A, Griffiths KM, Christensen H. Perceived barriers and facilitators to mental health help-seeking in young people: a systematic review. BMC Psychiatry. 2010 Dec 30;10(1):113.

27. Marcus M, Westra H, Mobilizing Minds Research Group. Mental Health Literacy in Canadian Young Adults: Results of a National Survey. Can J Commun Ment Health. 2012 Apr;31(1):1–15.

28. McAllister A, Fritzell S, Almroth M, Harber-Aschan L, Larsson S, Burström B. How do macro-level structural determinants affect inequalities in mental health? – a systematic review of the literature. Int J Equity Health. 2018 Dec 6;17(1):180.

29. Fleury MJ, Ngamini Ngui A, Bamvita JM, Grenier G, Caron J. Predictors of Healthcare Service Utilization for Mental Health Reasons. Int J Environ Res Public Health. 2014 Oct;11(10):10559–86.

30. Faber SC, Osman ,Muna, and Williams MT. Access to mental health care in Canada. Int J Ment Health. 2023 Jul 3;52(3):312–34.

31. Colonna C. Immigrant, Refugee, and Racialized Populations: Social Determinants of Health in Canada (2016 Census). 2019 Feb 22 [cited 2025 Apr 21]; Available from: https://policycommons.net/artifacts/11342405/immigrant-refugee-ethnocultural-and-racialized-populations-and-the-social-determinants-of/12231421/

32. Lai DWL, Li L, Daoust GD. Factors Influencing Suicide Behaviours in Immigrant and Ethno-Cultural Minority Groups: A Systematic Review. J Immigr Minor Health. 2017 Jun;19(3):755–68.

33. Faber SC, Williams MT, Metzger IW, MacIntyre MM, Strauss D, Duniya CG, et al. Lions at the gate: How weaponization of policy prevents people of colour from becoming professional psychologists in Canada. Can Psychol Psychol Can. 2023;64(4):335–5

34. Pang N, Nagata JM, Testa A, Ganson KT. Mapping eating disorders in adolescents and young adults: an investigation of geographic distribution and access to care in Ontario, Canada. Journal of eating disorders. 2024 Sep 9;12(1):136.

550

Formatted: Normal

**Response to Reviewer for Manuscript**: Sociodemographic and Mental Health Predictors of Mental Health Service Use Across Provider Types

**Reviewer #1**

**Reviewer Comment**: Title: I suggest the title to be revised to "Mental Health Predictors of Mental Health Service Use Across Provider Types".

**Author Response:** Thank you for the suggestion. We have opted to use the original title, "Sociodemographic and Mental Health Predictors of Mental Health Service Use Across Provider Types" as it more accurately reflects the scope of the study. In addition to mental health characteristics, our analysis includes a range of sociodemographic variables (e.g., age, gender, income, education, Indigenous identity, and immigration status) as key predictors.

**Reviewer Comment**: Abstract: Remove the last part of methodology.

**Author Response:** Thank you for the suggestion. We have removed the last part of methodology from the abstract.

**Reviewer Comment**: Abstract: Revise the results section in abstract for presenting the regression analysis.

**Author Response:** The results in the abstract have been edited when presenting the regression analysis and now reads: *In the adjusted regression models (2019–2020), women had higher odds of using family doctors (AOR = 1.21, 95% CI: 1.05–1.39) and social workers (1.19, 1.02–1.40) and lower odds of psychiatrists (AOR = 0.66, 95% CI:0.55–0.79) than men. Adults 65+ had greater odds of family-doctor use (AOR = 4.82, 95% CI: 3.59–6.47) and lower odds of psychologist (AOR = 0.33, 95% CI: 0.24–0.45) and social-worker use (AOR = 0.21, 95% CI:0.16–0.29) than ages 12–17. Post-secondary education (vs less than secondary school) was associated with higher psychologist use (AOR = 1.83, 95% CI 1.12–2.98). Higher income (≥$80,000 vs <$20,000) was associated with lower psychiatrist use (AOR = 0.66, 95% CI 0.50–0.86). Non-Indigenous respondents more often used psychologists (AOR = 1.58, 95% CI: 1.13–2.23), and respondents who are not a visible minority more often used family doctors (AOR = 1.37, 95% CI:1.06–1.77).*

**Reviewer Comment**: Introduction: Adding reference for middle of the second paragraph.

**Author Response:** Thank you for the suggestion. We have added a reference for the middle of the second paragraph.

**Reviewer Comment**: Provide a research gap and problem statement.

**Author Response:** We have revised this section to provide a cleaer research gap and problem statement. This section now reads as: *A limitation in this research however is that most studies aggregate all types of mental health providers into one variable limiting the examination of differences in who accesses which services and under which circumstances. As a result, we lack nuanced understanding of how sociodemographic*

*factors shape engagement with specific provider types, such as psychologists or social workers, whose roles and funding structures differ significantly from psychiatrists and family doctors. Furthermore, few studies have examined national level trends over time in provider specific mental health service use limiting our ability to assess how patterns have shifted in response to evolving service landscapes, policy changes, or public attitudes towards mental health. To address these gaps, this study focuses on examining how sociodemographic and mental health characteristics are associated with mental health service use across four provider types (family doctors, psychiatrists, psychologists, and social workers) and trends in their use over time in Canada. By disaggregating service use by provider type, this research aims to uncover distinct access patterns across sociodemographic groups and inform efforts to promote equity in mental health care.*

**Reviewer Comment:** Design of the study need to be mentioned the first.

**Author Response:** We now open the Methods with a clear statement of design and data source and now reads: *We conducted a cross-sectional, population-based analysis using seven cycles (2007-2020) of the Canadian Community Health Survey (CCHS) public-use microdata. The CCHS is a nationally representative cross-sectional survey administered by Statistics Canada. The CCHS is a self-reported survey on health, healthcare utilization, and health determinants of the Canadian population aged 12 and above. The CCHS covers the population 12 years of age and over who live in Canada. Excluded from the CCHS are institutionalized residents, full-time members of the Canadian Armed Forces, residents of certain remote regions, or people living on reserves/other Indigenous settlements in the provinces. Altogether, these exclusions represent less than 3% of the target population. Sample sizes varied across cycles, and all analyses applied survey weights provided by Statistics Canada to ensure representativeness of the Canadian population. This study summarized trends of provider specific mental health access across 2007–2020 and provider specific logistic regressions were restricted to 2019–2020 because it was the most recent cycle and contained key predictors specified a priori for multivariable regression. See Appendix 1 for details of variable availability and wording between CCHS cycles.*

**Reviewer Comment:** Inclusion and Exclusion Criteria not mentioned.

**Author Response:** Inclusion and exclusion criteria was added on the CCHS. This now reads: *The CCHS covers the population 12 years of age and over who live in Canada. Excluded from the CCHS are institutionalized residents, full-time members of the Canadian Armed Forces, residents of certain remote regions, or people living on reserves/other Indigenous settlements in the provinces. Altogether, these exclusions represent less than 3% of the target population.*

**Reviewer Comment:** Which type of logistic regression were used? Why? How?

**Author Response:** We clarified that we used survey-weighted multivariable logistic regression fitted separately for each provider and now reads: *To identify factors associated with provider-specific mental health service we fit four separate, survey-weighted multivariable logistic regression models using the 2019-2020 cycle of the data for each provider type (family doctor, psychiatrist, psychologist, and social worker).*

**Reviewer Comment:** Univariate and multivariate logistic regression analysis not performed, why?

**Author Response:** We did not perform univariate screening instead each provider-specific outcome was modeled with the prespecified multivariable set to avoid data-driven selection, preserve comparability across providers, and prevent exclusion of non-significant yet confounding covariates. Additionally, because key sociodemographic and health factors are correlated and rarely operate in isolation (e.g., income, education, racialization), multivariable models are required to estimate adjusted associations. This approach aligns with methodological guidance that discourages univariable screening in favor of theory-driven adjustment.

**Reviewer Comment:** Explain the logistic regression analysis in detail in the statistical analysis.

**Author Response:** We have added more detail into this statistical analysis and now reads as: *An exploratory descriptive analysis was conducted to examine trends in mental health service use across provider types over time (2007-2020). To identify factors associated with provider-specific mental health service we fit four separate, survey-weighted multivariable logistic regression models using the 2019-2020 cycle of the data for each provider type (family doctor, psychiatrist, psychologist, and social worker). The models access the relationship between the use of a specific provider adjusting for all other covariates. Each model included the same a priori covariates listed above to keep models comparable. Outcomes were binary (any past-12-month contact with the provider: yes/no). We modeled providers separately because respondents may see multiple providers and access differs for various reasons including pathways and financing. A single multinomial model would also impose inappropriate mutual exclusivity. Two-tailed tests with a threshold of α = 0.05 were used for all statistical tests. All analyses were conducted using R statistical software and survey weights were applied to account for the complex sampling design of the CCHS and to be representative of the Canadian population.*

**Reviewer Comment:** Presenting the results in the table 1 not enough indicated; Revise the comments for logistic regression.

**Author Response:** Thank you for this suggestion. We revised the Results to add clear, in-text interpretation of Table 1. We now summarize the key statistically significant findings in four short paragraphs organized by provider and include a brief non-findings paragraph to for simplicity. We also strengthened the Table 1 caption and notes.

**Reviewer Comment:** Discussion: Looks good.

**Author Response:** Thank you

**Reviewer Comment:** Conclusion: Looks good.

**Author Response:** Thank you

**Reviewer Comment:** References: Looks good.

**Author Response:** Thank you

**Reviewer Comment:** Figures and tables: Figures looks good.

**Author Response:** Thank you

**Reviewer Comment:** Some more issues should be considered necessary for publication: Suggestions for future studies also be mentioned.

**Author Response:** Thank you for this suggestion. We have a added a section on future directions for research that reads: *To build on these findings, future research should focus on using longitudinal or linked survey administrative data to map sequences of care, switching between providers, and duration/intensity of treatment. Similarly, methods that focus on concurrent mental health service provider use would be valuable allowing for the use of multinomial models and to better understanding how individuals are accessing the mental health care system. Equity should be a focus allowing exploration on intersectional effects across gender, age, income, education, racialization, immigration, Indigenous identity, and urbanicity. Likewise, further exploration is needed on needs and barriers including coverage, out-of-pocket costs, wait times, language, and providers availability. Lastly, qualitative research with equity seeking groups can illuminate preferences, experiences, and structural barriers that quantitative data alone may miss.*

**Reviewer Comment:** Please provide at least two related strengths for MS.

**Author Response:** Thank you for this suggestion. We have added a strength section that reads: *This study has several strengths. First, it leverages the Canadian Community Health Survey across seven cycles (2007–2020), yielding nationally representative estimates and trends, with Statistics Canada's sampling procedures and survey weights enhancing generalizability. Similarly, we use survey-weighted and fit provider-specific models (family doctor, psychiatrist, psychologist, social worker) allowing for a nuanced view of provider specific mental health care. Furthermore, the multivariable models are a priori and equity-focused, adjusting for correlated sociodemographic and health factors to explore variables related to policy.*

**Reviewer #2**

**Reviewer Comment:** There are numerous grammatical errors and awkward sentence constructions. This research will addresses key knowledge gaps... should be corrected to This research addresses key knowledge gaps....Consider thorough proofreading or language editing to improve readability and professionalism.

**Author Response:** Thank you for reviewing this manuscript. We have gone through the manuscript for proofreading to improve readability.

**Reviewer Comment:** The abstract is too long and includes excessive methodological detail. Condense the abstract and emphasize key findings and implications more succinctly.

**Author Response:** We have edited to abstract to be more concise and focus on key findings.

**Reviewer Comment:** You mention that logistic regressions were only done for 2019–2020 due to differences in variable measurement across survey cycles. However, you do not provide details or references showing which variables changed and how. Add an appendix table comparing variable definitions across cycles.

**Author Response:** Thank you for this suggestion. We have added Appendix 1: Variable Definitions and Availability Across CCHS Cycles (2007–2020), which lists each construct and the availability of the variable in each cycle and any changes to the variable. Reasons for conducting logistic regressions on the 2019-2020 cycle include visible minority only being available in the 2019-2020 cycle, indigenous identity first appearing n the 2015-2016 cycle, and household income being measured differently prior to 2013. We also added a sentence in Methods directing readers to Appendix 1 for further details on the variables and the CCHS cycles.

**Reviewer Comment:** The exclusion of provider overlap in the logistic models is a serious limitation. Many people access multiple providers. Discuss this more explicitly, and consider sensitivity analyses or multinomial models if feasible.

**Author Response:** We agree that the concurrent use is important. We now state explicitly in the results and limitations that outcomes were modeled separately as any contact per provider. For example, in the results we have added: *Because outcomes were modeled separately the estimates below should be interpreted as provider-specific associations.* And in the limitation we have added: *Likewise, given we did not model concurrent use across providers the results do not capture substitution, sequencing, or intensity of multi-provider care. Additionally, the CCHS lacks questions on visit timing, referral pathways, and number of contacts, limiting our ability to characterize multi-provider pathways. Furthermore, we did not have measures of symptom severity, diagnosis, functional impairment, prior treatment, local provider supply, wait times, out-of-pocket costs, or insurance and EAP coverage. We also lacked information on language, travel time, and referral practices. The lack of these factors does not provide a full picture of mental health service use and the omission of these factors could confound associations if they correlate with both with both sociodemographic characteristics and provider use. Because of this our findings should be interpreted as adjusted associations rather than causal effects. Future studies with linked longitudinal data and methods that explicitly accommodate overlap is needed.*
We considered the suggested sensitivity analyses and multinomial models but did not implement them for methodological and practical reasons. For example, multinomial models required many overlap categories and with small cell counts this will lead to instability under the complex survey design.

**Reviewer Comment:** Table 1 is dense and hard to interpret. Consider moving odds ratios and confidence intervals to supplemental material and highlighting only significant findings in the main text. You could benefit from a heatmap-style table or bolding to visually distinguish significant results.

**Author Response:** Thank you for this helpful suggestion. We retained the full numeric Table 1 to preserve exact effect sizes and confidence intervals. To improve readability, we bolded all statistically significant AORs additionally we have updated the results section to focus on each model to ease with readability. These changes to Table 1 make it easier to read while maintaining transparency.

**Reviewer Comment:** The manuscript references Figures 1 and 2, but they are not embedded or summarized effectively in the results. Embed or summarize the main trends and include captions that explain anomalies (2017 drop).

**Author Response:** Thank you for this suggestion we have edited the text to better summarize the main trends. This section now reads for figure 1: *Between 2007 and 2020, family doctors consistently accounted for the highest proportion of mental health service use in Canada in every cycle with over half of service users responding to having accessed a family doctor for mental health care (See Figure 1). Across all cycles, the rank order of providers was stable and the pattern over the years are relatively stable, with only modest year-to-year variation The proportion of people who accessed care through psychologists and social workers showed a gradual increase over time, while the proportion of visits to psychiatrists remained relatively unchanged over time. A one-year divergence appears in 2017, with a temporary spike for psychologist and dips slightly for the other providers but by the next cycle (2019) the pattern returns with psychologist and social workers converging at similar levels. We interpret 2017 as single cycle fluctuation rather than a break in trend. Estimates are survey-weighted and reflect that provider contacts are not mutually exclusive*
And for figure 2 the description now reads: *When examining weighted estimates of the number of Canadians accessing care, similar trends were observed (See Figure 2). The total number of individuals seeing family doctors for mental health reasons far exceeded those accessing psychologists, psychiatrists, or social workers. However, all provider types showed increases in absolute numbers over time reflecting increased mental service use overtime. Notably, a temporary drop in service use was observed in 2017 with a rebound by 2019 across all provider types, potentially reflecting data collection limitations in that survey cycle. Given each cycle of the CCHS has different weight calibrations and sample allocations, we believe this is a cycle level fluctuation rather than a structural change to mental health care use.*

**Reviewer Comment:** The discussion covers many findings but often lacks depth in interpretation. Provide more nuance on why certain sociodemographic factors (e.g., education, income) are predictive of use for certain providers. Integrate more Canadian-specific policy recommendations and barriers (wait times, geographic access, EAP programs).

**Author Response:** Thank you for this suggestion we have added throughout to the discussion section. For example we have added more on age as predictor: *Furthermore, these age patterns likely also reflect differences in care continuity and navigation. Older adults have stronger ties to a usual family doctor and more multimorbidity which makes*

*primary care a natural entry point for mental and physical concerns. Younger adults in contrast more often have access to private insurance and Employee Assistance Programs (EAP) for counselling which may lower the threshold to see psychologist and social workers.*
Costs: *Specifically, in Canada psychologist and social workers are not universally covered and access often depends on employers benefits, EAPs, or private insurance which commonly cap the number of sessions. By contrast, visits to family doctors and psychiatrist are publicly insured which may explain a shift towards these providers among lower income groups.*
Geographic disparities: *Furthermore, these disparities are also shaped by geography and provider supply. For example, mental health care providers are often concentrated in large urban centre resulting in longer travel times and indirect costs for rural communities to accessing care.*

Canadian-specific policy recommendations*: Several actions could improve equitable access to mental health services in Canada. First, expanding publicly funded, evidence-based psychotherapy would reduce the role of financial barriers. Second, integrating mental health care providers into primary care teams by streamlining referrals, provided shared care can result in shortened wait times and improved continuity in care. Equity can be further strengthened through Indigenous-led services, cultural-safety standards, language interpretation, and navigation supports for newcomers and racialized communities. Furthermore, private insurance and EAPs need to be modernized to ensure adequate session coverage. Lastly, further investment needs to be made in community-based mental health care including walk-in counselling, youth hubs, peer and Indigenous led programs and system navigation would expand timely service and reduce avoidable reliance on emergency and specialist services.*

**Reviewer Comment:** The limitations section is too brief. Elaborate on limitations of self-report data, cross-sectional design, and unobserved confounders (e.g., provider availability, wait times, severity of need).

**Author Response:** Thank you for this suggestion. We have updated the limitations section, and it now reads as: *This study has several limitations. First, mental health service use was based on self-report and may be subject to recall or reporting biases. Second, only service use within the past year was captured without accounting for frequency, duration, or perceived adequacy of care. Third, the regression models only used cross-sectional data from 2019–2020. This is due to an inability to combine data from different cycles together as predictor variables were measured differently across various cycles of the survey. This means that logistic regression results are not able to describe longer-term patterns. Additionally, service use was measured separately for each provider type, without information on concurrent or integrated care. Likewise, given we did not model concurrent use across providers the results do not capture substitution, sequencing, or intensity of multi-provider care. Additionally, the CCHS lacks questions on visit timing, referral pathways, and number of contacts, limiting our ability to characterize multi-provider pathways. Furthermore, we did not have measures of symptom severity, diagnosis, functional impairment, prior treatment, local provider*

*supply, wait times, out-of-pocket costs, or insurance and EAP coverage. We also lacked information on language, travel time, and referral practices. The lack of these factors does not provide a full picture of mental health service use and the omission of these factors could confound associations if they correlate with both with both sociodemographic characteristics and provider use. Because of this our findings should be interpreted as adjusted associations rather than causal effects. Future studies with linked longitudinal data and methods that explicitly accommodate overlap is needed.*

**Reviewer Comment:** While you mention Statistics Canada data, the Data Availability Statement is vague. Include exact dataset names, links to access procedures, and R code availability (if possible).

**Author Response:** We have revised the data availability statement to include information and new reads:

*Data Access The data that support the findings of this study are provided by Statistics Canada microdata files. This study used the Canadian Community Health Survey (CCHS) Public Use Microdata Files (PUMF) for the following cycles: 2007–2008, 2009–2010, 2011–2012, 2013–2014, 2015–2016, 2017–2018, and 2019–2020.*

*The dataset names are as follows:*

1. *Canadian Community Health Survey, 2007-2008: Annual Component*
2. *Canadian Community Health Survey, 2009-2010: Annual Component*
3. *Canadian Community Health Survey, 2011-2012: Annual Component*
4. *Canadian Community Health Survey, 2013-2014: Annual Component*
5. *Canadian Community Health Survey, 2015-2016: Annual Component*
6. *Canadian Community Health Survey, 2017-2018: Annual Component*
7. *Canadian Community Health Survey, 2019-2020: Annual Component*

*Files were accessed through ODESI via the University of Toronto. The R scripts used for data preparation, survey-weighted analyses, and figure generation are available from the corresponding author upon reasonable request for research purposes.*

**Reviewer Comment:** Include OR values in the abstract for the most important findings. Improve consistency in terminology — e.g., switch between visible minority and non-visible minority inconsistently.

**Author Response:** Thank you for this suggestion. We have edited to the abstract to include OR values and have gone through the manuscript for consistency in terminology.
