# Supplementary material for: Sociodemographic and mental health predictors of mental health service use across provider types
Source: PLoS One. 2026 May 18;21(5):e0326556. doi: 10.1371/journal.pone.0326556 (PMC13183218; doi:10.1371/journal.pone.0326556)
Supplement: Attachment — Submitted filename: Response to Reviewers.docx [file pone.0326556.s002.docx]

**Response to Reviewer for Manuscript**: Sociodemographic and Mental Health Predictors of Mental Health Service Use Across Provider Types

**Reviewer #1**

**Reviewer Comment**: Title: I suggest the title to be revised to “Mental Health Predictors of Mental Health Service Use Across Provider Types”.

**Author Response:** Thank you for the suggestion. We have opted to use the original title, “Sociodemographic and Mental Health Predictors of Mental Health Service Use Across Provider Types” as it more accurately reflects the scope of the study. In addition to mental health characteristics, our analysis includes a range of sociodemographic variables (e.g., age, gender, income, education, Indigenous identity, and immigration status) as key predictors.

**Reviewer Comment**: Abstract: Remove the last part of methodology.

**Author Response:** Thank you for the suggestion. We have removed the last part of	methodology from the abstract.

**Reviewer Comment**: Abstract: Revise the results section in abstract for presenting the regression analysis.

**Author Response:** The results in the abstract have been edited when presenting the regression analysis and now reads: *In the adjusted regression models (2019–2020), women had higher odds of using family doctors (AOR = 1.21, 95% CI: 1.05–1.39) and social workers (1.19, 1.02–1.40) and lower odds of psychiatrists (AOR = 0.66, 95% CI:0.55–0.79) than men. Adults 65+ had greater odds of family-doctor use (AOR = 4.82, 95% CI: 3.59–6.47) and lower odds of psychologist (AOR = 0.33, 95% CI: 0.24–0.45) and social-worker use (AOR = 0.21, 95% CI:0.16–0.29) than ages 12–17. Post-secondary education (vs less than secondary school) was associated with higher psychologist use (AOR = 1.83, 95% CI 1.12–2.98). Higher income (≥$80,000 vs <$20,000) was associated with lower psychiatrist use (AOR = 0.66, 95% CI 0.50–0.86). Non-Indigenous respondents more often used psychologists (AOR = 1.58, 95% CI: 1.13–2.23), and respondents who are not a visible minority more often used family doctors (AOR = 1.37, 95% CI:1.06–1.77).*

**Reviewer Comment**: Introduction: Adding reference for middle of the second paragraph.

**Author Response:** Thank you for the suggestion. We have added a reference for the	middle of the second paragraph.

**Reviewer Comment**: Provide a research gap and problem statement.

**Author Response:** We have revised this section to provide a cleaer research gap and problem statement. This section now reads as: *A limitation in this research however is that most studies aggregate all types of mental health providers into one variable limiting the examination of differences in who accesses which services and under which circumstances. As a result, we lack nuanced understanding of how sociodemographic factors shape engagement with specific provider types, such as psychologists or social workers, whose roles and funding structures differ significantly from psychiatrists and family doctors. Furthermore, few studies have examined national level trends over time in provider specific mental health service use limiting our ability to assess how patterns have shifted in response to evolving service landscapes, policy changes, or public attitudes towards mental health. To address these gaps, this study focuses on examining how sociodemographic and mental health characteristics are associated with mental health service use across four provider types (family doctors, psychiatrists, psychologists, and social workers) and trends in their use over time in Canada. By disaggregating service use by provider type, this research aims to uncover distinct access patterns across sociodemographic groups and inform efforts to promote equity in mental health care.*

**Reviewer Comment:** Design of the study need to be mentioned the first.

**Author Response:** We now open the Methods with a clear statement of design and data source and now reads: *We conducted a cross-sectional, population-based analysis using seven cycles (2007-2020) of the Canadian Community Health Survey (CCHS) public-use microdata. The CCHS is a nationally representative cross-sectional survey administered by Statistics Canada. The CCHS is a self-reported survey on health, healthcare utilization, and health determinants of the Canadian population aged 12 and above. The CCHS covers the population 12 years of age and over who live in Canada. Excluded from the CCHS are institutionalized residents, full-time members of the Canadian Armed Forces, residents of certain remote regions, or people living on reserves/other Indigenous settlements in the provinces. Altogether, these exclusions represent less than 3% of the target population. Sample sizes varied across cycles, and all analyses applied survey weights provided by Statistics Canada to ensure representativeness of the Canadian population. This study summarized trends of provider specific mental health access across 2007–2020 and provider specific logistic regressions were restricted to 2019–2020 because it was the most recent cycle and contained key predictors specified a priori for multivariable regression. See Appendix 1 for details of variable availability and wording between CCHS cycles.*

**Reviewer Comment:** Inclusion and Exclusion Criteria not mentioned.

**Author Response:** Inclusion and exclusion criteria was added on the CCHS. This now reads: *The CCHS covers the population 12 years of age and over who live in Canada. Excluded from the CCHS are institutionalized residents, full-time members of the Canadian Armed Forces, residents of certain remote regions, or people living on reserves/other Indigenous settlements in the provinces. Altogether, these exclusions represent less than 3% of the target population.*

**Reviewer Comment:** Which type of logistic regression were used? Why? How?

**Author Response:** We clarified that we used survey-weighted multivariable logistic regression fitted separately for each provider and now reads: *To identify factors associated with provider-specific mental health service we fit four separate, survey-weighted multivariable logistic regression models using the 2019-2020 cycle of the data for each provider type (family doctor, psychiatrist, psychologist, and social worker).*

**Reviewer Comment:** Univariate and multivariate logistic regression analysis not performed, why?

**Author Response:** We did not perform univariate screening instead each provider-specific outcome was modeled with the prespecified multivariable set to avoid data-driven selection, preserve comparability across providers, and prevent exclusion of non-significant yet confounding covariates. Additionally, because key sociodemographic and health factors are correlated and rarely operate in isolation (e.g., income, education, racialization), multivariable models are required to estimate adjusted associations. This approach aligns with methodological guidance that discourages univariable screening in favor of theory-driven adjustment.

**Reviewer Comment:** Explain the logistic regression analysis in detail in the statistical analysis.

**Author Response:** We have added more detail into this statistical analysis and now reads as: *An exploratory descriptive analysis was conducted to examine trends in mental health service use across provider types over time (2007-2020). To identify factors associated with provider-specific mental health service we fit four separate, survey-weighted multivariable logistic regression models using the 2019-2020 cycle of the data for each provider type (family doctor, psychiatrist, psychologist, and social worker). The models access the relationship between the use of a specific provider adjusting for all other covariates. Each model included the same a priori covariates listed above to keep models comparable. Outcomes were binary (any past-12-month contact with the provider: yes/no). We modeled providers separately because respondents may see multiple providers and access differs for various reasons including pathways and financing. A single multinomial model would also impose inappropriate mutual exclusivity. Two-tailed tests with a threshold of α = 0.05 were used for all statistical tests. All analyses were conducted using R statistical software and survey weights were applied to account for the complex sampling design of the CCHS and to be representative of the Canadian population.*

**Reviewer Comment:** Presenting the results in the table 1 not enough indicated; Revise the comments for logistic regression.

**Author Response:** Thank you for this suggestion. We revised the Results to add clear, in-text interpretation of Table 1. We now summarize the key statistically significant findings in four short paragraphs organized by provider and include a brief non-findings paragraph to for simplicity. We also strengthened the Table 1 caption and notes.

**Reviewer Comment:** Discussion: Looks good.

**Author Response:** Thank you

**Reviewer Comment:** Conclusion: Looks good.

**Author Response:** Thank you

**Reviewer Comment:** References: Looks good.

**Author Response:** Thank you

**Reviewer Comment:** Figures and tables: Figures looks good.

**Author Response:** Thank you

**Reviewer Comment:** Some more issues should be considered necessary for publication: Suggestions for future studies also be mentioned.

**Author Response:** Thank you for this suggestion. We have a added a section on future directions for research that reads: *To build on these findings, future research should focus on using longitudinal or linked survey administrative data to map sequences of care, switching between providers, and duration/intensity of treatment. Similarly, methods that focus on concurrent mental health service provider use would be valuable allowing for the use of multinomial models and to better understanding how individuals are accessing the mental health care system. Equity should be a focus allowing exploration on intersectional effects across gender, age, income, education, racialization, immigration, Indigenous identity, and urbanicity. Likewise, further exploration is needed on needs and barriers including coverage, out-of-pocket costs, wait times, language, and providers availability. Lastly, qualitative research with equity seeking groups can illuminate preferences, experiences, and structural barriers that quantitative data alone may miss.*

**Reviewer Comment:** Please provide at least two related strengths for MS.

**Author Response:** Thank you for this suggestion. We have added a strength section that reads: *This study has several strengths. First, it leverages the Canadian Community Health Survey across seven cycles (2007–2020), yielding nationally representative estimates and trends, with Statistics Canada’s sampling procedures and survey weights enhancing generalizability. Similarly, we use survey-weighted and fit provider-specific models (family doctor, psychiatrist, psychologist, social worker) allowing for a nuanced view of provider specific mental health care. Furthermore, the multivariable models are a priori and equity-focused, adjusting for correlated sociodemographic and health factors to explore variables related to policy.*

**Reviewer #2**

**Reviewer Comment:** There are numerous grammatical errors and awkward sentence constructions. This research will addresses key knowledge gaps... should be corrected to This research addresses key knowledge gaps....Consider thorough proofreading or language editing to improve readability and professionalism.

**Author Response:** Thank you for reviewing this manuscript. We have gone through the	manuscript for proofreading to improve readability.

**Reviewer Comment:** The abstract is too long and includes excessive methodological detail. Condense the abstract and emphasize key findings and implications more succinctly.

**Author Response:** We have edited to abstract to be more concise and focus on key	findings.

**Reviewer Comment:** You mention that logistic regressions were only done for 2019–2020 due to differences in variable measurement across survey cycles. However, you do not provide details or references showing which variables changed and how. Add an appendix table comparing variable definitions across cycles.

**Author Response:** Thank you for this suggestion. We have added Appendix 1: Variable Definitions and Availability Across CCHS Cycles (2007–2020), which lists each construct and the availability of the variable in each cycle and any changes to the variable. Reasons for conducting logistic regressions on the 2019-2020 cycle include visible minority only being available in the 2019-2020 cycle, indigenous identity first appearing n the 2015-2016 cycle, and household income being measured differently prior to 2013. We also added a sentence in Methods directing readers to Appendix 1 for further details on the variables and the CCHS cycles.

**Reviewer Comment:** The exclusion of provider overlap in the logistic models is a serious limitation. Many people access multiple providers. Discuss this more explicitly, and consider sensitivity analyses or multinomial models if feasible.

 **Author Response:** We agree that the concurrent use is important. We now state explicitly	in the results and limitations that outcomes were modeled separately as any contact per	provider. For example, in the results we have added: *Because outcomes were modeled	separately the estimates below should be interpreted as provider-specific associations.*	And in the limitation we have added: *Likewise, given we did not model concurrent use	across providers the results do not capture substitution, sequencing, or intensity of multi-	provider care. Additionally, the CCHS lacks questions on visit timing, referral pathways,	and number of contacts, limiting our ability to characterize multi-provider pathways.	Furthermore, we did not have measures of symptom severity, diagnosis, functional	impairment, prior treatment, local provider supply, wait times, out-of-pocket costs, or	insurance and EAP coverage. We also lacked information on language, travel time, and	referral practices. The lack of these factors does not provide a full picture of mental	health service use and the omission of these factors could confound associations if they	correlate with both with both sociodemographic characteristics and provider use.	Because of this our findings should be interpreted as adjusted associations rather than	causal effects. Future studies with linked longitudinal data and methods that explicitly	accommodate overlap is needed.*

We considered the suggested	sensitivity analyses and multinomial models but did not	implement them for methodological and practical reasons. For example, multinomial	models required many overlap categories and with small cell counts this will lead to	instability under the complex survey design.

**Reviewer Comment:** Table 1 is dense and hard to interpret. Consider moving odds ratios and confidence intervals to supplemental material and highlighting only significant findings in the main text. You could benefit from a heatmap-style table or bolding to visually distinguish significant results.

**Author Response:** Thank you for this helpful suggestion. We retained the full numeric Table 1 to preserve exact effect sizes and confidence intervals. To improve readability, we bolded all statistically significant AORs additionally we have updated the results section to focus on each model to ease with readability. These changes to Table 1 make it easier to read while maintaining transparency.

**Reviewer Comment:** The manuscript references Figures 1 and 2, but they are not embedded or summarized effectively in the results. Embed or summarize the main trends and include captions that explain anomalies (2017 drop).

**Author Response:** Thank you for this suggestion we have edited the text to better summarize the main trends. This section now reads for figure 1: *Between 2007 and 2020, family doctors consistently accounted for the highest proportion of mental health service use in Canada in every cycle with over half of service users responding to having accessed a family doctor for mental health care (See Figure 1). Across all cycles, the rank order of providers was stable and the pattern over the years are relatively stable, with only modest year-to-year variation The proportion of people who accessed care through psychologists and social workers showed a gradual increase over time, while the proportion of visits to psychiatrists remained relatively unchanged over time. A one-year divergence appears in 2017, with a temporary spike for psychologist and dips slightly for the other providers but by the next cycle (2019) the pattern returns with psychologist and social workers converging at similar levels. We interpret 2017 as single cycle fluctuation rather than a break in trend. Estimates are survey-weighted and reflect that provider contacts are not mutually exclusive*
And for figure 2 the description now reads: *When examining weighted estimates of the number of Canadians accessing care, similar trends were observed (See Figure 2). The total number of individuals seeing family doctors for mental health reasons far exceeded those accessing psychologists, psychiatrists, or social workers. However, all provider types showed increases in absolute numbers over time reflecting increased mental service use overtime. Notably, a temporary drop in service use was observed in 2017 with a rebound by 2019 across all provider types, potentially reflecting data collection limitations in that survey cycle. Given each cycle of the CCHS has different weight calibrations and sample allocations, we believe this is a cycle level fluctuation rather than a structural change to mental health care use.*

**Reviewer Comment:** The discussion covers many findings but often lacks depth in interpretation. Provide more nuance on why certain sociodemographic factors (e.g., education, income) are predictive of use for certain providers. Integrate more Canadian-specific policy recommendations and barriers (wait times, geographic access, EAP programs).

**Author Response:** Thank you for this suggestion we have added throughout to the discussion section. For example we have added more on age as predictor: *Furthermore, these age patterns likely also reflect differences in care continuity and navigation. Older adults have stronger ties to a usual family doctor and more multimorbidity which makes primary care a natural entry point for mental and physical concerns. Younger adults in contrast more often have access to private insurance and Employee Assistance Programs (EAP) for counselling which may lower the threshold to see psychologist and social workers.*

Costs: *Specifically, in Canada psychologist and social workers are not universally covered and access often depends on employers benefits, EAPs, or private insurance which commonly cap the number of sessions. By contrast, visits to family doctors and psychiatrist are publicly insured which may explain a shift towards these providers among lower income groups.*

Geographic disparities: *Furthermore, these disparities are also shaped by geography and provider supply. For example, mental health care providers are often concentrated in large urban centre resulting in longer travel times and indirect costs for rural communities to accessing care.*

Canadian-specific policy recommendations*: Several actions could improve equitable access to mental health services in Canada. First, expanding publicly funded, evidence-based psychotherapy would reduce the role of financial barriers. Second, integrating mental health care providers into primary care teams by streamlining referrals, provided shared care can result in shortened wait times and improved continuity in care. Equity can be further strengthened through Indigenous-led services, cultural-safety standards, language interpretation, and navigation supports for newcomers and racialized communities. Furthermore, private insurance and EAPs need to be modernized to ensure adequate session coverage. Lastly, further investment needs to be made in community-based mental health care including walk-in counselling, youth hubs, peer and Indigenous led programs and system navigation would expand timely service and reduce avoidable reliance on emergency and specialist services.*

**Reviewer Comment:** The limitations section is too brief. Elaborate on limitations of self-report data, cross-sectional design, and unobserved confounders (e.g., provider availability, wait times, severity of need).

**Author Response:** Thank you for this suggestion. We have updated the limitations section, and it now reads as: *This study has several limitations. First, mental health service use was based on self-report and may be subject to recall or reporting biases. Second, only service use within the past year was captured without accounting for frequency, duration, or perceived adequacy of care. Third, the regression models only used cross-sectional data from 2019–2020. This is due to an inability to combine data from different cycles together as predictor variables were measured differently across various cycles of the survey. This means that logistic regression results are not able to describe longer-term patterns. Additionally, service use was measured separately for each provider type, without information on concurrent or integrated care. Likewise, given we did not model concurrent use across providers the results do not capture substitution, sequencing, or intensity of multi-provider care. Additionally, the CCHS lacks questions on visit timing, referral pathways, and number of contacts, limiting our ability to characterize multi-provider pathways. Furthermore, we did not have measures of symptom severity, diagnosis, functional impairment, prior treatment, local provider supply, wait times, out-of-pocket costs, or insurance and EAP coverage. We also lacked information on language, travel time, and referral practices. The lack of these factors does not provide a full picture of mental health service use and the omission of these factors could confound associations if they correlate with both with both sociodemographic characteristics and provider use. Because of this our findings should be interpreted as adjusted associations rather than causal effects. Future studies with linked longitudinal data and methods that explicitly accommodate overlap is needed.*

**Reviewer Comment:** While you mention Statistics Canada data, the Data Availability Statement is vague. Include exact dataset names, links to access procedures, and R code availability (if possible).

**Author Response:** We have revised the data availability statement to include	information and new reads:

*Data Access The data that support the findings of this study are provided by Statistics Canada microdata files. This study used the Canadian Community Health Survey (CCHS) Public Use Microdata Files (PUMF) for the following cycles: 2007–2008, 2009–2010, 2011–2012, 2013–2014, 2015–2016, 2017–2018, and 2019–2020.*

*The dataset names are as follows:*

*1.	Canadian Community Health Survey, 2007-2008: Annual Component*

*2.	Canadian Community Health Survey, 2009-2010: Annual Component*

*3.	Canadian Community Health Survey, 2011-2012: Annual Component*

*4.	Canadian Community Health Survey, 2013-2014: Annual Component*

*5.	Canadian Community Health Survey, 2015-2016: Annual Component*

*6.	Canadian Community Health Survey, 2017-2018: Annual Component*

*7.	Canadian Community Health Survey, 2019-2020: Annual Component*

*Files were accessed through ODESI via the University of Toronto. The R scripts used for data preparation, survey-weighted analyses, and figure generation are available from the corresponding author upon reasonable request for research purposes.*

**Reviewer Comment:** Include OR values in the abstract for the most important findings. Improve consistency in terminology — e.g., switch between visible minority and non-visible minority inconsistently.

**Author Response:** Thank you for this suggestion. We have edited to the abstract to	include OR values and have gone through the manuscript for consistency in terminology.
