# Supplementary material for: Sociodemographic and mental health predictors of mental health service use across provider types
Source: PLoS One. 2026 May 18;21(5):e0326556. doi: 10.1371/journal.pone.0326556 (PMC13183218; doi:10.1371/journal.pone.0326556)
Supplement: Attachment — Submitted filename: Response_to_Reviewers_auresp_2.docx [file pone.0326556.s004.docx]

**Response to Reviewer for Manuscript**: Sociodemographic and Mental Health Predictors of Mental Health Service Use Across Provider Types

**Reviewer #3**

**Reviewer Comment:** There are 2 occasions in which the authors stated that the study is cross sectional, whereas it is retrospective. This needs to be corrected

**Author Response:** Thank you for this comment. We agree that the manuscript should more clearly distinguish between the cross-sectional nature of the CCHS data and the retrospective nature of our analysis. We have revised the manuscript to describe the study as a retrospective secondary analysis of cross-sectional survey data where appropriate. For instance, the methods section now reads: *“We conducted a retrospective secondary analysis of seven cycles, using seven cycles (2007-2020) of the Canadian Community Health Survey (CCHS) public-use microdata.”*

**Reviewer #4**

**Reviewer Comment:** The current manuscript leverages large national Canada data over the years 2007-2020 to explore mental healthcare help-seeking by type of provider and illuminates potential disparities in regard to access to care. This is an important area of research with great implications for public health messaging and other interventions aimed at improving access. The current manuscript is a revision of a previous submission. I was not a reviewer of the previous draft, and so this is my first encounter with this work. Overall while the topic is important and the data sufficient to meet the research question, in my opinion there are several areas in need of revision.

**Author Response:** Thank you your review of this manuscript. Below, we address each of your comments in detail.

**Reviewer Comment:** A major issue that needs to be addressed in the Introduction and the Discussion is the impact of the COVID-19 pandemic on access to mental healthcare, particularly with regard to telehealth. The fact that all data precede what constituted a massive shift in service provision is a substantial limitation. The authors need to justify how and why these data are still relevant to the practice of mental healthcare in 2026.

**Author Response:** Thank you for this comment. We agree that the COVID-19 pandemic led to substantial changes in mental health service delivery, particularly through the expansion of telehealth, and that our data (2007–2020) largely reflect pre-pandemic patterns of care. While our study captures pre-pandemic service use, we argue that this data provides an important baseline for understanding structural patterns in access to different provider types, many of which (e.g., funding models and provider distribution) continue to shape access in the post-pandemic era.

We have added to the background section: *“The data used in this study predate the COVID-19 pandemic, which led to rapid expansion of virtual mental health care and changes in service delivery across provider types. While these changes may have changed some patterns of mental health care access, pre-pandemic data remain important for establishing baseline trends and identifying structural inequities in mental health service use. Understanding these baseline patterns is essential for interpreting how access may have changed in the post-COVID-19 pandemic context.”*

We have added to discussion section: *“An important consideration in interpreting these findings is that the data reflect pre-pandemic patterns of mental health service use. The COVID-19 pandemic led to a multitude of changes in mental health care including the expansion of virtual care and telehealth services, which may have altered access to different provider types.^23^ For example, virtual care may have reduced some geographic barriers while potentially introducing new challenges related to technology access, digital literacy, and privacy. Despite these changes, many structural features of the Canadian mental health system such as differences in public versus private funding and provider availability remain largely unchanged. Because of this the patterns observed in this study likely reflect underlying systemic dynamics that continue to shape access to care. Likewise, these findings provide an important baseline for understanding how mental health service use has evolved in the post-COVID-19 pandemic era.”*

**Reviewer Comment:** The assent/consent process for youth aged 12-18 needs to be explained.

**Author Response:** Data collection for the CCHS is conducted by Statistics Canada, which obtains informed consent from all participants. We have added: *“For respondents aged 12–17 years, participation requires parental or guardian consent in addition to youth assent, in accordance with Statistics Canada data collection procedures.”*

**Reviewer Comment:** Were response options for the outcome variable limited to the 4 provider types? Were there others? Or was it a free response that then was coded by researchers?

**Author Response:** The outcome variable was based on predefined response categories in the Canadian Community Health Survey (CCHS), where respondents were asked whether they had seen or talked to specific types of health professionals about their mental health in the past 12 months. The CCHS includes multiple provider categories (i.e., family doctors, psychiatrists, psychologists, social workers, nurses, and other health professionals).

This section now reads:*“Response options in the CCHS are provided as predefined categories for different provider types (i.e., family doctor, psychiatrist, psychologist, social worker, nurse, and other health professionals), and respondents may select multiple providers. This analysis focused on the four most common providers for mental health care (family doctor, psychiatrist, psychologist, and social work) to ensure interpretability and comparability across cycles. Each provider was coded as a separate variable as respondents could see more than one provider.”*

**Reviewer Comment:** How was “Visible minority” defined?

**Author Response:** In the CCHS, this variable is defined by Statistics Canada in accordance with the Employment Equity Act. The Employment Equity Act defines visible minorities as "persons, other than Aboriginal peoples, who are non-Caucasian in race or non-white in colour". The visible minority population consists mainly of the following groups: South Asian, Chinese, Black, Filipino, Arab, Latin American, Southeast Asian, West Asian, Korean and Japanese. We have added this clarification to the Methods section: “*Visible minority status is defined by Statistics Canada, in accordance with the Employment Equity Act, as persons, other than Indigenous peoples, who are non-Caucasian in race or non-white in colour, based on self-identification.”*

**Reviewer Comment:** I did not see any sample descriptives. This basic information is needed for readers to contextualize the findings.

**Author Response:** We have added a table with sample descriptives and the accompanying text: *“Table 1 presents the weighted characteristics of respondents who reported consulting a mental health professional in the past 12 months. The sample was predominantly women (65.9%) and individuals aged 18–49 years. Most respondents reported higher household incomes (≥$80,000; 60.1%) and post-secondary education (87.1%). The majority identified as non-visible minorities (86.2%) and non-immigrants (83.7%), with 5.0% identifying as Indigenous. Most participants reported good or very good general (67.5%) and mental health (62.2%).”*

**Reviewer Comment:** The authors interpret the pattern of service use over the years as “relatively stable.” is this a subjective observation or were differences between years tested? The latter would strengthen the manuscript.

**Author Response:** Thank you for this comment. We agree that the phrasing could imply formal statistical testing. We have revised the manuscript to clarify that interpretations of stability are based on descriptive trends rather than formal tests of differences across years. This clarification has been added to both the Results and Limitations sections.

The results now read: *“Across all cycles, the rank order of providers was stable and the patterns of service use appeared relatively stable over time based on descriptive trends, although no formal statistical tests of differences across years were conducted.”*

In the limitations we added: *“Furthermore, we did not formally test differences in service use across survey years a result interpretations of temporal trends are based on descriptive patterns.”*

**Reviewer Comment:** Table 1 is extremely hard to read and is not formatted following APA guidelines

**Author Response:** Thank you for this comment. We have reformatted Table 2 to improve readability and align with APA guidelines. Specifically, we combined adjusted odds ratios and confidence intervals into a single column, removed p-value columns, and simplified the table structure.

**Reviewer Comment:** Several assertions are made in the Discussion with no accompanying reference.

**Author Response:** Thank you for the comment we have added reference throughout where appropriate.
